# PEGylated Liposomal Resveratrol Induces Region-Specific Redox Modulation Without Behavioral or Neurotrophin Recovery in a VPA Model of Autism

**DOI:** 10.1007/s12031-026-02574-1

**Published:** 2026-08-01

**Authors:** Victória Linden de Rezende, Maria Fernanda Pedro Ebs, Flávia da Silva Daros, Maiara de Aguiar da Costa, Sofia Januário Bolan, Germano Lapa Viana, Leonardo Pelegrini, Nathan de Souza Colonetti, Maria Júlia Ferro Spillere, Giseli da Silva Souza, Rafaela de Sousa Anastácio, Ellen De-Pieri, Júlio César Claudino dos Santos, Ricardo Andrez Machado de Ávila, Alexandre Gonçalves Dal-Bó, Cinara Ludvig Gonçalves

**Affiliations:** 1 Laboratory of Experimental Neurology, Graduate Program in Health Sciences, Universidade do Extremo Sul Catarinense (UNESC), Criciúma, SC Brazil; 2 Laboratory of Experimental Pathophysiology, Graduate Program in Health Sciences, Universidade do Extremo Sul Catarinense (UNESC), Criciúma, SC Brazil; 3 Advanced Polymer Processing Laboratory, Graduate Program in Materials Science and Engineering, Universidade do Extremo Sul Catarinense (UNESC), Criciúma, SC Brazil; 4 Graduate Program in Neurology and Neuroscience, Fluminense Federal University (UFF), Niterói, Rio de Janeiro Brazil

**Keywords:** Autism Spectrum Disorder, Valproic Acid, Liposomes, Resveratrol, Oxidative Stress

## Abstract

Autism Spectrum Disorder (ASD) is associated with neuroinflammation and oxidative stress that disrupt neurodevelopmental processes. Resveratrol (RSV) is a polyphenol with antioxidant and anti-inflammatory properties, but its poor bioavailability limits therapeutic use. This study investigated whether PEGylated liposomes encapsulating RSV (LipRSV) could modulate oxidative and behavioral alterations in a valproic acid (VPA)-induced rat model of ASD. PEGylated LipRSV were synthesized, characterized as hemocompatible, and administered to offspring from postnatal day (PND) 6 to 27. Behavioral tests included developmental milestones, olfactory discrimination, negative geotaxis, open field, and three-chamber social interaction. Oxidative stress markers and neurotrophins (BDNF, NGF) were quantified in the following brain regions: hippocampus, hypothalamus, striatum, cerebellum, frontal and posterior cortex. LipRSV exerted limited and region-dependent redox effects, reducing ROS levels only in the hippocampus, cerebellum, and posterior cortex, while failing to improve behavioral outcomes and being associated with unfavorable neurochemical alterations, including reduced BDNF and NGF levels. Importantly, LipRSV also induced behavioral deficits, redox imbalance, and neurochemical alterations in control animals, indicating treatment-related effects in the absence of VPA exposure. LipRSV modulated oxidative stress in a region-dependent manner without improving behavioral deficits in the VPA model and was associated with reduced BDNF and NGF levels. This study provides evidence that modulation of oxidative stress alone is insufficient to rescue behavioral phenotypes in the VPA model of ASD. These findings highlight the partial neurochemical efficacy and translational limitations of this formulation, suggesting the need for optimized nanocarrier design and dosing strategies in ASD pharmacotherapy.

**Supplementary Information:**

The online version contains supplementary material available at https://doi.org/10.1007/s12031-026-02574-1.

## Introduction

Autism Spectrum Disorder (ASD) is a complex neurodevelopmental condition characterized by impaired social communication, restricted interests, repetitive behaviors, and atypical sensory processing (APA, 2022). ASD has shown an exponential rise in prevalence, now affecting approximately 1 in 31 children (Maenner et al. 2023) and is widely recognized as a multifactorial disorder resulting from complex interactions between genetic and environmental factors (Lord et al. 2018). Heritability estimates range from 50% to 90%, underscoring the strong genetic contribution to its pathogenesis (Griesi-Oliveira and Sertié 2017; Kainer et al. 2023). Environmental influences, including maternal immune activation (Gardner et al. 2025; Meyer 2014) and prenatal exposure to neurotoxic agents such as valproic acid (VPA) (Roullet et al. 2013; Xu et al. 2025), have also been implicated in idiopathic ASD (Varghese et al. 2017). Pathophysiologically, neuroinflammation and oxidative stress are key mechanisms underlying the disorder (Ahmad et al. 2018; Frasch et al. 2023; Komada and Nishimura 2022).

Neuroinflammation is primarily mediated by activated microglia and astrocytes that release pro-inflammatory cytokines, thereby exacerbating central nervous system (CNS) inflammation (Ahmad et al. 2018). Increased microglial activity and elevated cytokine levels have been demonstrated in patients with ASD (Komada and Nishimura 2022). In parallel, oxidative stress contributes to pathogenesis through an imbalance between pro-oxidant and antioxidant systems, promoting cellular injury and immune dysfunction. Individuals with ASD show reduced antioxidant defenses, such as glutathione depletion, accompanied by increased lipid peroxidation and redox disequilibrium (Kashyap et al. 2018; Lima Giacobbo et al. 2019). These processes compromise neurogenesis, neuronal migration, and synapse formation (Komada and Nishimura 2022). Microglial dysfunction further impairs synaptic pruning, maintaining immature excitatory synapses and disrupting excitatory–inhibitory balance, a hallmark of ASD-related cognitive and behavioral deficits (Toscano et al. 2021). Excessive cytokine activity, including IL-1β, IL-6, and TNF-α, amplifies these alterations (Hughes et al. 2023). Moreover, inflammation dysregulates neurotrophins such as BDNF and NGF, which are essential for neuronal growth and plasticity via TrkA/B/C and p75^NTR^ signaling pathways (Numakawa and Kajihara 2023; Skaper 2012, 2018). Together, the inflammatory and oxidative imbalance contributes to the structural and functional brain alterations characteristic of ASD (Galvez-Contreras et al. 2017; Lucaci et al. 2022).

The treatment of ASD remains challenging due to its etiological heterogeneity and the limited efficacy of current pharmacological interventions (Masi et al. 2017; Siegel and Beaulieu 2012). The blood-brain barrier (BBB) restricts drug penetration into the CNS, limiting the bioavailability of therapeutic agents (Alajangi et al. 2022). Nanoparticle-based delivery systems have emerged as a promising approach for brain-targeted therapy, provided they exhibit favorable physicochemical and safety profiles (Masserini 2013). Among these systems, liposomes are biocompatible vesicles consisting of a lipid bilayer surrounding an aqueous core (Liu et al. 2022). Resveratrol (RSV), a natural polyphenol with antioxidant and anti-inflammatory activity, has been extensively investigated for its neuroprotective potential (Berman et al. 2017; Rauf et al. 2017). However, its poor solubility, rapid metabolism, and low bioavailability hinder clinical translation (Franciosoa et al. 2014). Encapsulation of RSV in PEGylated liposomes has emerged as a promising strategy to enhance its stability and systemic circulation, with potential to improve CNS availability.

Despite extensive evidence linking oxidative stress to ASD, few studies have explored liposomal or nanoparticle-based delivery of RSV in this context. Given this gap, the present study investigated the effects of PEGylated liposomes encapsulating RSV (LipRSV) on behavioral, oxidative, and neurochemical parameters in a VPA-induced rat model of autism.

## Materials and Methods

### Liposomes Preparation

The Liposomes encapsulating RSV were prepared using the reverse-phase evaporation method as previously described (Feuser et al., 2021b; Kroetz et al., 2019). Two formulations were produced: pure phosphatidylcholine (PC) liposomes and mixed liposomes composed of PC and C22PEG900GlcNAc in a 70:30 w/w ratio. For both formulations, RSV (1.0 mg/mL) was incorporated into the hydrophobic phase using chloroform. The lipid components (6 mg/mL PC or 6 mg/mL PC + C22PEG900GlcNAc) were dissolved in 10 mL of chloroform, followed by the addition of 200 µL phosphate-buffered saline (PBS, 10 mM). The mixture was sonicated for 4 min at 25 °C using an LGI-LUC sonicator (São Paulo, Brazil; 180 W maximum power) to form a homogeneous and opalescent reverse micelle dispersion.

The organic solvent was evaporated under reduced pressure at 30 °C using a rotary evaporator (IKA RV 10, Staufen, Germany) operating at 180 rpm and 380 mbar, producing a viscous organogel. This procedure yielded both pure (PC) and PEGylated (PC + C22PEG900GlcNAc) liposomes, with or without RSV encapsulation. The resulting dispersions were filtered through nylon membranes (0.45 μm pore size, Whatman, GE Healthcare Life Sciences) to remove aggregates and stored at room temperature (20–25 °C) until use. The liposomal formulation was prepared using phosphatidylcholine-based PEGylated liposomes, with a total lipid concentration of 6 mg/mL. RSV was incorporated into the liposomal system at a final concentration of 1 mg/mL. Thus, the 6 mg/mL concentration refers to the lipid phase/liposomal components, whereas the 1 mg/mL concentration refers specifically to RSV in the final LipRSV formulation. Because the encapsulation efficiency of RSV was not determined in the present study, the exact proportion of encapsulated versus non-encapsulated RSV could not be established. Therefore, the RSV concentration reported here refers to the nominal total RSV concentration in the liposomal formulation.

The physicochemical characteristics of the liposomes, including particle size, polydispersity, and zeta potential, were later analyzed as previously described (de Castro et al. 2019; dos Santos et al. 2018; Cardoso dos Santos et al. 2021).

### Liposomes Characterization

The physicochemical parameters of the liposomal formulations were determined to evaluate their stability and uniformity. Measurements included hydrodynamic diameter (2RH), polydispersity index (PDI) and zeta potential (ZP) (Table 1), which were obtained using dynamic light scattering (DLS) with a NanoBrook Omni analyzer (Brookhaven Instruments Corporation, Holtsville, NY, USA).

**Table 1:** Dynamic light scattering (DLS) characterization of synthesized liposomes

Samples	2R_H_ (nm)	PDI	ZP (mV)	ZP (mV)^b^
Phosphatidylcholine Liposome	249.1	0.207	-55.4±0.8	-53,2±0.5
Phosphatidylcholine Liposome + Resveratrol	319.5	0.190	-54.7±0.2	-48.5±0.4
Phosphatidylcholine Liposome + PEG	321.3	0.177	-56.0±0.9	-52.6±0.5
Phosphatidylcholine Liposome + PEG + Resveratrol	343.0	0.255	-51.3±0.5	-47.4±0.2

^b^ Zeta Potential Analysis After 30 Days

Hydrodynamic diameter (2RH), polydispersity index (PDI), and zeta potential (ZP) of liposomal formulations with and without resveratrol (RSV) were measured using dynamic light scattering (DLS) on the day of synthesis and after 30 days of storage. All formulations exhibited nanometric sizes. The incorporation of RSV led to an increase in particle diameter with minimal changes in PDI, except for the phosphatidylcholine + PEG + RSV formulation, which showed higher size heterogeneity. Zeta potential values remained highly negative (|ZP| > 50 mV), indicating good colloidal stability. Data are presented as mean ± standard deviation (SD)

All analyses were performed at 25 °C after appropriate dilution of the samples in ultrapure water to avoid multiple scattering effects. Each measurement was carried out in triplicate (*n*= 3), and results were expressed as mean ± standard deviation (SD). Data reliability and reproducibility were confirmed according to previously described protocols described (de Castro et al. 2019; dos Santos et al. 2018; Cardoso dos Santos et al. 2021).

The amphiphilic compound N-acetil-β-D-glucosaminil-PEG900-docosanato (C22PEG900GlcNAc − 1509 g/mol) was synthesized by esterification of N3-PEG900-OH with docosanoic acid, followed by a “click chemistry” reaction with a modified carbohydrate, as described in the literature. Purification was performed by silica gel chromatography, with a yield of 67%. Liposomes containing only phosphatidylcholine (PC) or mixtures of PC and C22PEG900GlcNAc (70:30, w/w), with or without RSV, were prepared using the reverse-phase evaporation method. RSV was incorporated in the organic phase with chloroform, followed by sonication and solvent removal under reduced pressure, forming an organogel. The formulations (6 mg/mL of liposomes and 1.0 mg/mL of RSV) were filtered and stored at room temperature.

Physicochemical characterization included particle size distribution, polydispersity index (PDI), and zeta potential (ZP) analysis by dynamic light scattering (DLS), with measurements performed in triplicate (Table 1).

### Hemocompatibility Test

All experiments were conducted in accordance with the Research Ethics Committee (CEP) of the Universidade do Extremo sul Catarinense, which approved them before the start of the experimental procedures, under authorization no. 3,344,689. Human erythrocytes were provided by three healthy volunteers. The total blood (3 mL) was centrifuged for 10 min at 1,500 rpm to remove the supernatant. Then, three washes were performed with saline solution (0.9% NaCl) and centrifuged three times to remove other cells and traces of plasma. After the third wash, the supernatant was discarded, and the erythrocytes were diluted with 1.5 mL of saline solution (0.9% NaCl).

After obtaining the erythrocytes, hemolytic activity was assessed by spectrophotometry. The selected compounds for this assay were incubated at different concentrations (6 mg/mL, 3.0 mg/mL, 1.5 mg/mL, and 0.75 mg/mL of the samples, and 0.06 mg/mL, 0.03 mg/mL, 0.015 mg/mL and 0.0075 mg/mL of Alendronate) and incubated with 50 µL of human erythrocytes resuspended in 950 µL of saline solution. The incubation time was one hour at 37 °C, under continuous agitation at 100 rpm. After the incubation period (1 h), the solution was centrifuged for five minutes (10,000 rpm) at room temperature. Then, 100 µL of the sample supernatant was transferred to a 96-well plate. The hemocompatibility percentage was evaluated by spectrophotometry. Additionally, the absorbance was measured at 540 nm using a SpectraMax Plus 384 microplate reader. The positive control (distilled water) and the negative control (saline solution) were also performed. The results were expressed as the hemocompatibility percentage, obtained from the equation below:

$$\begin{array}{c}Hemocompatibility\;rate\;\left(\%\right)\\=\left(Dt-Dnc\right)\times100/\left(Dpc-Dnc\right)\end{array}$$

Where Dt = absorbance of the test sample; Dnc = absorbance of the negative control; Dpc = absorbance of the positive control. The assays were performed in triplicate (Amin and Dannenfelser 2006).

As mentioned above, methodological reliability was confirmed using positive (distilled water) and negative (0.9% saline) controls, yielding expected hemolysis values of 100% and < 1%, respectively, with a defined hemolysis threshold of 5%. The hemolytic potential of the formulations was interpreted according to the ASTM F756-17 standard, in which samples are classified as non-hemolytic when hemolysis is < 2%, slightly hemolytic when hemolysis is between 2% and 5%, and hemolytic when hemolysis is > 5%. Therefore, formulations presenting hemolysis values below 5% were considered acceptable for further biological evaluation. PEG-containing liposomes, with or without RSV, demonstrated high hemocompatibility, maintaining hemolysis below 4% at the highest concentration and below 1% at lower concentrations. In contrast, the PC + PEG liposome formulation exhibited a dose-dependent increase in hemolysis, reaching 100% at the highest concentration and decreasing to approximately 10% at the lowest (*p* < 0.05 vs. other formulations). Notably, the addition of RSV to PEG + PC liposomes significantly mitigated hemolytic toxicity, maintaining hemolysis below 3% across all concentrations, comparable to PEG-only formulations (Supplementary data).

### Animal Model and Ethics Statement

*Wistar* rats from the breeding colony at the Universidade do Extremo Sul Catarinense (UNESC), Brazil, were housed four times per cage under controlled conditions of temperature (22 ± 1 °C), relative humidity (45–55%) and light-dark cycle (12:12 p.m., light on at 6 a.m.). Rat chow (standard diet for laboratory animals - NUVILAB CR-1^®^, Brazil) and filtered water were available *ad libitum*. All experimental procedures involving animals were conducted following international recommendations for the care and use of laboratory animals, as well as the guidelines of the Brazilian Society of Neurosciences and Behavior for Animal Care. This study was submitted for evaluation by the Ethics Committee on the Use of Animals (CEUA) of UNESC under protocol number 07/2023.

**Fig. 1:**
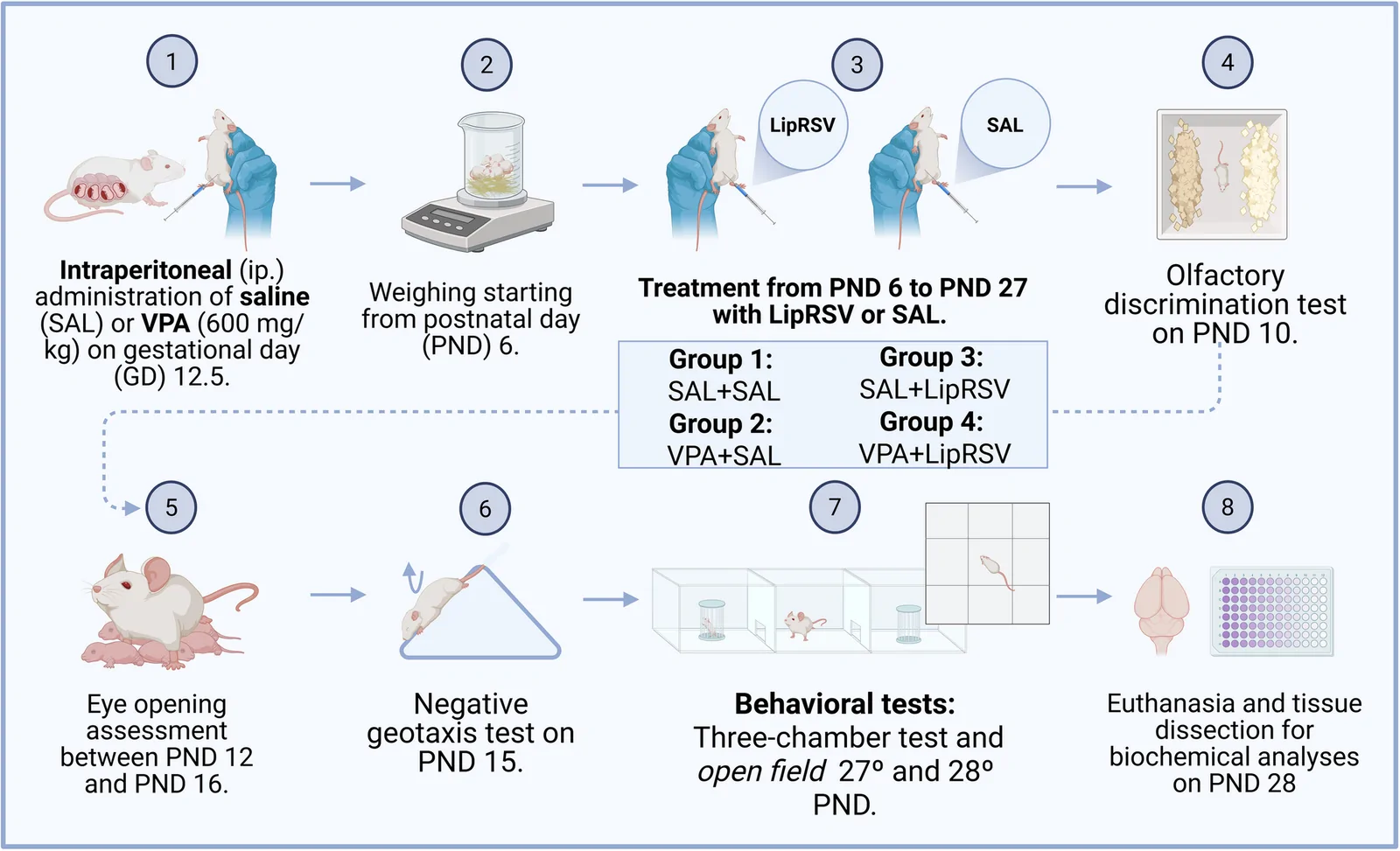
Experimental Design. Experimental timeline of prenatal VPA exposure and postnatal LipRSV treatment. Pregnant rats received intraperitoneal injections of saline (SAL) or VPA (600 mg/kg) on gestational day 12.5 (GD 12.5). Offspring were weighed from postnatal day (PND) 6 and treated with LipRSV or SAL from PND 6 to PND 27. Behavioral assessments included olfactory discrimination (PND 10), eye opening (PND 12–16), negative geotaxis (PND 15), three-chamber social test and open field (PND 27–28). Animals were euthanized on PND 28 for tissue collection and biochemical analysis

To obtain the necessary number of animals for the experiment, eight *Wistar* females were mated with eight *Wistar* males (1:1, individual cages) after checking the estrous cycle adapted from Marcondes and collaborators (2002). To encourage mating, bedding (wood shavings) used by the males was placed in the females’ cages. After 24 h of adding the litter, the estrous cycle of eight rats was determined by collecting vaginal cells with 80 µL of sterile 0.9% saline (SAL) solution, which were examined on microscope slides. After confirmation of estrus cells, females were paired with a male in a clean cage for one night. The morning after mating, a vaginal wash was performed to check the presence of sperm. When sperm were found, it was considered gestational day 0.5 (GD 0.5), and the female was called a dam. The dams were housed in cages containing two to four females until GD 18, when they were isolated for the birth of the offspring. On GD12.5, the dams were weighed and were divided into two groups: one received an intraperitoneal (i.p.) injection of 600 mg/kg of VPA (Sigma-Aldrich, St. Louis, MO), (VPA-exposed group), while control animals received an equivalent volume of saline (SAL) adjusted according to body weight (0.09% NaCl) via i.p., (SAL-exposed group). On GD 18.5, the dams were isolated for the birth of the offspring. For this project, only male offspring were used. The entire experimental design is presented in Fig. 1.

### Animals Treatment

Based on the results of the hemocompatibility test, the compound with the best safety profile for *in vivo* application was selected. Accordingly, LipRSV, composed of phosphatidylcholine-based PEGylated liposomes with a total lipid concentration of 6 mg/mL and RSV at a final concentration of 1 mg/mL, was selected for animal treatment. LipRSV was administered intraperitoneally during the experimental treatment period. The injection volume was adjusted according to body weight using a proportional dosing rule based on a reference volume of 100 µL for a 30 g animal. Based on the final RSV concentration of 1 mg/mL, this administration regimen corresponded to a nominal RSV dose of approximately 3.33 mg/kg. This dose is also close to RSV regimens previously reported in VPA-induced animal models of ASD, in which RSV was administered at approximately 3.6 mg/kg for 12–13 days (Fontes-Dutra et al., 2018; Hirsch, et al., 2018; Bambini-Junior et al. 2014).

### Postnatal Growth and Development

#### Behavioral Tests

The behavioral tests were performed in a particular room, during the light cycle, at a constant temperature of 22 ± 1 °C, with water and food *ad libitum*. All the animals were acclimatized in the experimental room for 1 h before the tests. It is important to mention that the technician in charge of conducting the behavioral tests did not know the animals’ experimental groups.

#### Olfactory Discrimination Test: Nest Seeking Behavior

On postnatal day 10 (PND10), male pups were subjected to the nest-seeking test to evaluate the functional maturation of the olfactory system. The experimental procedure was based on the protocol described by (Gregory and Pfaff 1971). Briefly, each animal was placed in a rat cage containing bedding with a familiar odor (from the home cage) on one end and sterilized odorless bedding on the opposite end. A 6 cm central zone was left clear of bedding to serve as the starting area. The pup was gently positioned in the center of the apparatus, facing away from the odor cue (i.e., facing the technician), and the time required for the animal to reach the familiar-scented bedding was recorded. The latency to reach the target was measured, with a maximum cutoff time of 60 s. The apparatus was cleaned with 70% alcohol, followed by 10% alcohol between each trial to prevent olfactory cue contamination. After the test, each pup was returned to its home cage.

#### Eye-Opening Evaluation

During the interval between PNDs 12 to 16, eye opening was monitored as an indicator of visual system maturation. The evaluation was performed once daily in pups born to dams exposed to SAL or VPA. Scoring was based on the method described by (Schneider and Przewłocki 2005), using a scale from 0 to 1: a score of 0 indicated that all pups had their eyes closed; 0.5 was assigned when at least one pup had opened eyes; and a score of 1 indicated that all pups had fully opened eyes.

#### Negative Geotaxis Test

On PND 15, the negative geotaxis test was performed to assess the maturation of vestibular and motor systems. The protocol was adapted from (Ruhela et al. 2019) and involved placing each pup on an inclined plane set at a 45° angle, with its head oriented downward. The latency to turn the body and rotate the head upward (against gravity) was recorded. If the pup failed to exhibit the geotactic reflex on the first attempt, the procedure was repeated up to three times, within a maximum time limit of 60 s.

#### Social Interaction

On PND 28, male rats underwent the three-chamber test, adapted from (Moy et al. 2004). This test is based on rodents’ natural tendency to investigate unfamiliar animals and intruders. The rats were placed in an apparatus consisting of three acrylic chambers (120 × 40 × 40 cm) connected by removable dividers (10 × 20 cm). Initially, the rat was placed in the central chamber for a 5-minute habituation period. After this, an unknown rat (Rat 1) was placed in a small cage in the left chamber, while the right chamber remained empty. The dividers were removed to allow the test rat to explore all three chambers freely for 10 min. Following this, the test rat was placed back in the central chamber, enclosed by dividers, and a second unknown rat (Rat 2) was introduced into the right chamber. The dividers were then removed to allow the test rat to explore the apparatus for another 10 min. After the test, the apparatus was cleaned using 70% alcohol, followed by 10% alcohol. The animal was then returned to its original cage. The following parameters were recorded: latency to first encounter with Rats 1 and 2, number of encounters with Rats 1 and 2 in each phase, interaction time with Rats 1 and 2, and time spent in each chamber (left and right).

#### Open Field

On PND 27 and 28, two hours after the three-chamber test on PND 28, rats were subjected to the open field test to assess exploratory and locomotor activity (Deacon 2006). The test, adapted from (Cheng et al. 2020), was conducted in a square arena (60 × 60 × 48 cm) with the floor divided into nine equal squares. The procedure consisted of two phases: train (habituation) and test. During the train phase, the animal was placed in a corner quadrant and allowed freely explore the apparatus for 5 min to promote habituation to the experimental environment. During this phase, the number of quadrant crossings with all four paws (indicative of locomotor activity) and the number of*rearing* episodes (standing on the hind limbs, indicative of exploratory activity) were recorded. At the end of this period, the animal was removed from the arena. The test phase was conducted on the following day, at the same time as the train phase. The animal was again placed in the arena under the same conditions and allowed to explore for 5 min. During this phase, the same behavioral parameters were recorded. After the behavioral test, the apparatus was cleaned with 70% alcohol, followed by 10% alcohol, and the animal was returned to its original cage.

### Biochemical Analysis

#### Oxidative Stress Parameters

##### Protein Analysis

The protein concentration in the homogenized samples was determined using bovine serum albumin as a standard, following the adapted protocol originally described by (Lowry et al. 1951). The Folin phenol reagent binds to the protein, resulting in a gradual reduction, indicated by a color change from yellow to blue. Absorbance was measured at 650 nm.

##### Catalase Analysis

For the determination of H_2_O_2_degradation, as adapted from (Aebi 1984), the decrease in absorbance at 240 nm was measured. Samples were exposed to H_2_O_2_ or buffer (blank control), and absorbance was recorded immediately at 240 nm in eight intervals ranging from 1 to 40 s. Results were expressed as µmols of H_2_O_2_ per mg of protein.

##### Superoxide Dismutase Analysis

SOD activity was assessed by measuring the inhibition of adrenaline auto-oxidation according to (Bannister and Calabrese 1987). A 96-well plate was used, where 10 µL of the sample was mixed with 180 µL of glycine buffer at 32 °C, followed by 5 µL of catalase. The endpoint was measured at 412 nm, and after adding 5 µL of adrenaline, readings were taken every 40 s for 20 min. The results were expressed as SOD Units per mg of protein (U/mg).

##### Sulfhydryl Analysis

The non-oxidized sulfhydryl (SH) groups in the sample were quantified according to (Aksenov and Markesbery 2001). The reduction of DTNB by non-oxidized thiols forms a yellow derivative (5-thio-2-nitrobenzoate), which was measured at 412 nm, providing the total thiol content in the sample. Carbonyl compounds in the proteins were quantified by precipitating proteins with 20% trichloroacetic acid, followed by resuspension in dinitrophenylhydrazine (DNPH). For the blank, proteins were resuspended in HCl.

##### Carbonyl Content Analysis

The carbonyl content was determined by absorbance at 370 nm, and the total carbonyl content was estimated by the difference in absorbance between DNPH-treated and HCl-treated samples, as described by (Reznick and Packer 1994).

##### 2’,7’ Dichlorofluorescein Analysis

The concentration of 2’,7’ dichlorofluorescein (DCF) was measured in homogenized brain samples. DCF diacetate was added to 80 µg protein aliquots, which were incubated at 37 °C for 30 min. Esterases cleave the acetyl group from DCF-DA, generating DCFH, which is then oxidized by reactive species to produce the fluorescent DCF. Fluorescence was measured at excitation and emission wavelengths of 480 nm and 535 nm, respectively. A calibration curve using standard DCF (0–10 µM) was created, and the results were expressed as pmol of DCF per mg of protein, following (LeBel et al. 1990).

#### Cytokines and Neurotrophins Analysis

The concentrations of TNF-α (Rat TNF-α ELISA Kit, Thermo Fisher Scientific, Cat # KRC3011),BDNF, and NGF (Rat BDNF DuoSet ELISA, R&D Systems, Cat# NBP3-42309; Rat beta-NGF DuoSet ELISA, R&D Systems, Cat# AF-556-NA) were analysed in the posterior cortex by enzyme-bound immunosorbent assay (ELISA) in a microplate photometer using commercial kits (Peprotech, São Paulo, Brazil). The results were expressed as picograms per milliliter (pg/mL).

### Statistical Analysis

The data were analyzed for normality using the Shapiro-Wilk test and for homogeneity using Levene’s test. For data that were normally distributed and homogeneous, parametric tests were used, specifically one-way ANOVA followed by Tukey’s post hoc test. When the data did not meet these assumptions, non-parametric tests were applied, namely the Kruskal-Wallis test followed by Dunn’s post hoc test. For comparisons between the SAL and VPA groups, Student’s t-test was used for normally distributed data, and the Mann-Whitney test was used for non-normally distributed data. Statistical significance was considered at *p* < 0.05. All analyses were performed using GraphPad Prism version 9.0.

## Results

### Behavioral Assessment

#### Neurodevelopmental Tests

**Fig. 2:**
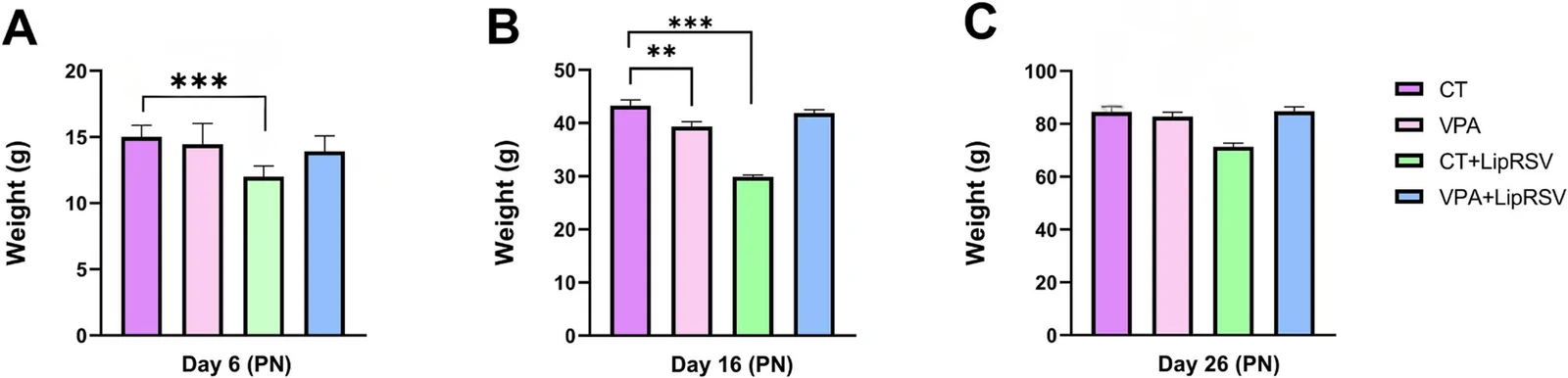
Effects of treatment with liposomes containing resveratrol (LipRSV) on the animals weight. The graphs show (**A**) weight on postnatal day (PND) 6, (**B**) weight on PND 16, and (**C**) weight on PND 26. Data are expressed as mean ± standard deviation (SD). Statistical analysis was performed using one-way ANOVA followed by Tukey’s post hoc test for multiple group comparisons. **p < 0.01, ***p < 0.0001. (*n* = 10 animals/group)

From PND 6 to day 26, all animals (*n* = 10 animals/group) were individually weighed and the data recorded. The data presented in Fig. 2A, B, and C represent the body weights of the pups on PND 6, 16, and 26, respectively. In Fig. 2A, the CT+LipRSV group shows a significantly lower body weight (***p < 0.0001) compared to the CT group. Figure 2B shows the average body weight on PND 16, where the VPA group presented significantly lower weight (**p < 0.01) compared to the CT group. Meanwhile, the CT+LipRSV group showed a significantly lower body weight compared to both the CT group (**p < 0.0001). In Fig. 2C, no significant alterations were observed.

**Fig. 3:**
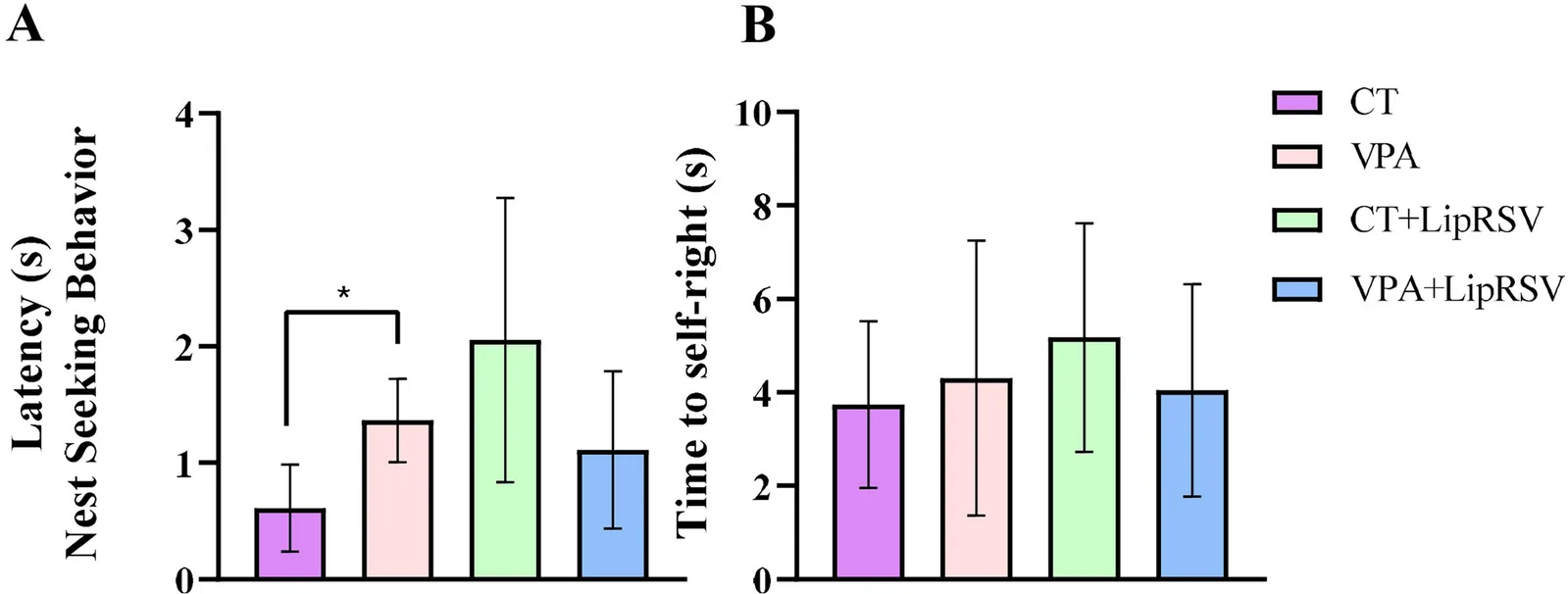
Effects of treatment with liposomes containing resveratrol (LipRSV) on neurodevelopmental parameters. The graphs show (**A**) latency in the nest-seeking test and (**B**) time to turn in the negative geotaxis test. The parameters were measured in seconds (s). Data are expressed as mean ± standard deviation (SD). Statistical analysis was performed using one-way ANOVA followed by Tukey’s post hoc test, with statistical significance considered at *p < 0.05. (*n* = 10 animals/group)

On PND 10, all animals underwent the olfactory discrimination test (*n* = 10 animals/group). The main parameter measured was latency, which refers to the time the animal takes to initiate and complete a specific action during the test (Fig. 3A). Latency to find the nest was significantly higher in the VPA group (*p < 0.05) compared to the CT group. In addition, the negative geotaxis test was performed to assess vestibular and/or proprioceptive function, as well as coordination in the animals. On postnatal day 15, animals were individually evaluated (*n* = 10 per group). The time each animal took to rotate to the opposite position, i.e., with its head facing upward, was recorded. The results showed no significant differences between the groups (Fig. 3B).

#### Open Field

**Fig. 4:**
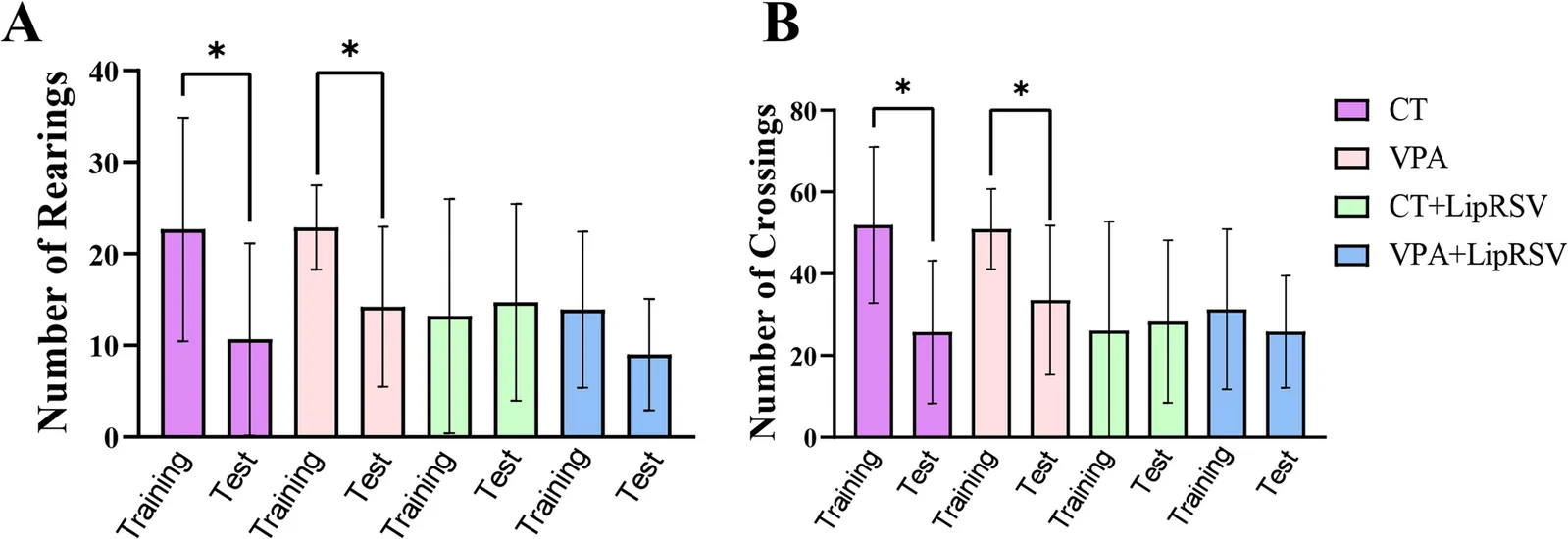
Effects of treatment with liposomes containing resveratrol (LipRSV) in the open field test. The graphs show (**A**) the number of rearings and (**B**) the number of crossings recorded during two test sessions. The experimental groups included control animals exposed and treated with saline (CT), animals exposed to valproic acid (VPA), CT animals treated with LipRSV (CT+LipRSV), and VPA animals treated with LipRSV (VPA+LipRSV). The first bar represents Train (PND 27), and the second represents Test (PND 28). Data are expressed as mean ± standard deviation of the mean (SMD), with *n* = 10 animals per group. Statistical analyses were performed by two-way ANOVA, considering *p* < 0.05 as the significance level. The symbol (*) indicates significant differences between trainings and test

The open field test was employed to assess exploratory behavior, locomotor activity, and habituation memory in juvenile rats (*n* = 10 per group) on PND 27 and 28. Behavioral parameters included the number of rearings (vertical exploratory movements) (Fig. 4A) and the number of crossings (horizontal activity) (Fig. 4B).

**Fig. 5:**
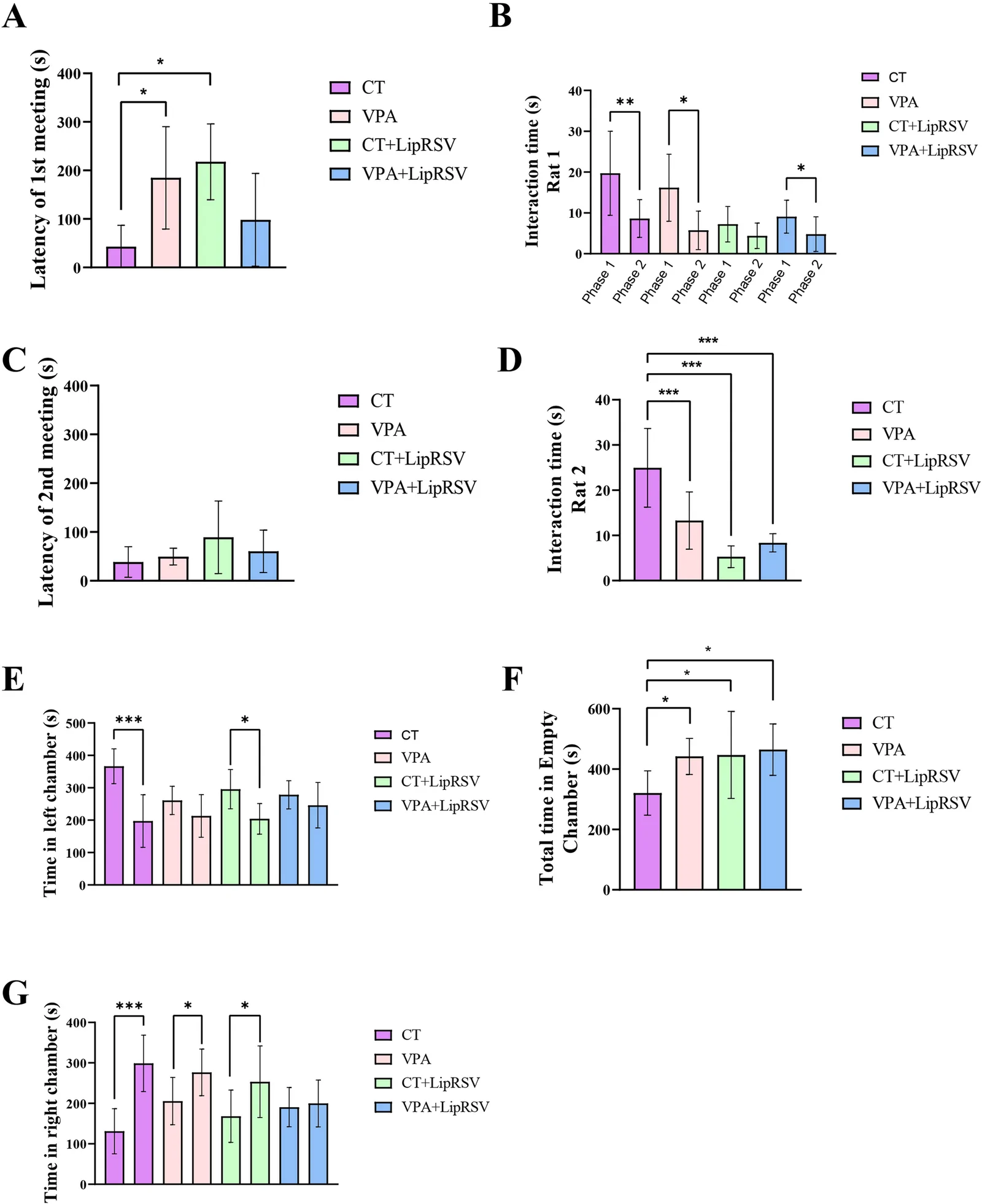
Effects of treatment with liposomes containing resveratrol (LipRSV) on social behavior in the three-chamber test. (**A**) latency to the first social approach, (**B**) interaction time during the sociability and novelty phases (Rat 1), (**C**) latency to the second approach, and (**D**) Interaction time with the novel social stimulus animal (rat 2). Panels (**E**), (**F**), and (**G**) represent the total time spent in the left chamber, the empty chamber, and the right chamber, respectively. Experimental groups included saline-treated controls (CT), valproic acid-exposed animals (VPA), CT animals treated with LipRSV (CT+LipRSV), and VPA animals treated with LipRSV (VPA+LipRSV). Data are expressed as mean ± standard deviation (SD), with *n* = 10 animals per group. Statistical analysis was performed using two-way ANOVA, with *p* < 0.05 considered statistically significant. The symbol (*) indicates statistically significant differences between groups or test phases. *p < 0.05, **p < 0.01, ***p < 0.001

There was a significant reduction (*p < 0.05) in the number of rearings on the test day in both CT and VPA groups when compared to the training day. However, no statistically significant differences were detected between the experimental groups on either day. Similarly, a significant decrease (*p < 0.05) in the number of crossings on the test day was observed in CT and VPA animals (Fig. 5B). No significant intergroup differences were found for this parameter. These results show that all experimental groups, regardless of VPA exposure or LipRSV treatment, exhibited a reduction in exploratory behavior over time, consistent with the process of habituation.

##### Three Chamber-Test

The three-chamber test is widely used to assess social behavior in rodents, allowing the investigation of two main aspects: sociability (the tendency to interact with a conspecific compared to an inanimate stimulus) and preference for social novelty (the ability to discriminate and prefer a novel individual over a familiar one). The test consists of two moments: the sociability phase (phase 1), in which the animal can interact with an unfamiliar conspecific, and the social novelty preference phase (phase 2), in which the animal chooses between a familiar animal and a novel one. In general, longer social interaction time and shorter latency to initiate interaction are interpreted as indicators of preserved social behavior. In the sociability phase, there was a significant increase (*p < 0.05) in the latency to the first interaction in the VPA and CT+LipRSV groups, compared to the CT group (Fig. 5A).

Regarding social interaction time, the CT group spent more time interacting with the social stimulus animal (rat 1) during the sociability phase (Fig. 5B) than during the social novelty phase (phase 2) (**p < 0.01). A similar pattern was observed in the VPA and VPA+LipRSV groups (*p < 0.05). There were no statistically significant differences between the groups in latency to the second interaction (Fig. 5C). In the evaluation of interaction with the novel social stimulus animal (rat 2) (Fig. 5D), all experimental groups (VPA, CT+LipRSV, and VPA+LipRSV) exhibited significantly reduced interaction time compared to the CT group (***p < 0.001). The experimental groups displayed partial preservation of social approach behavior, but impaired social novelty preference and altered exploratory social patterns.

About the time spent in the left chamber (Fig. 5E), there was a significant reduction in the social novelty phase (phase 2) in the CT group (***p < 0.001) and the CT+LipRSV group (*p < 0.05), with no significant differences between the other groups. The total time spent in the empty chamber (Fig. 5F) increased in the VPA, CT+LipRSV and VPA+LipRSV groups (* *p* < 0.05) in comparison to the CT group.

Regarding the time spent in the right chamber (Fig. 5G), the CT group had a longer time in the novelty phase (phase 2) when compared to the sociability phase (phase 1) (***p < 0.001), as did the VPA and CT+LipRSV groups (*p < 0.05). The data suggest that animals in the CT group exhibit a coordinated spatial response to social novelty; however, the experimental groups, particularly the VPA group, appear to display a less efficient exploration pattern, which is consistent with impaired social novelty processing.

### Oxidative Stress Parameters in Brain Areas

On PND 28, animals were euthanized, and the brain was removed and dissected into specific regions of interest: frontal cortex, hypothalamus, hippocampus, striatum, posterior cortex, and cerebellum. To investigate the neuroprotective effects of LipRSV, oxidative stress markers were quantified in these brain regions. The analyses included CAT and SOD enzymatic activities, quantification of total sulfhydryl groups, and measurement of ROS production using the DCF (2’,7’-dichlorofluorescein) assay.

#### Frontal Cortex

**Fig. 6:**
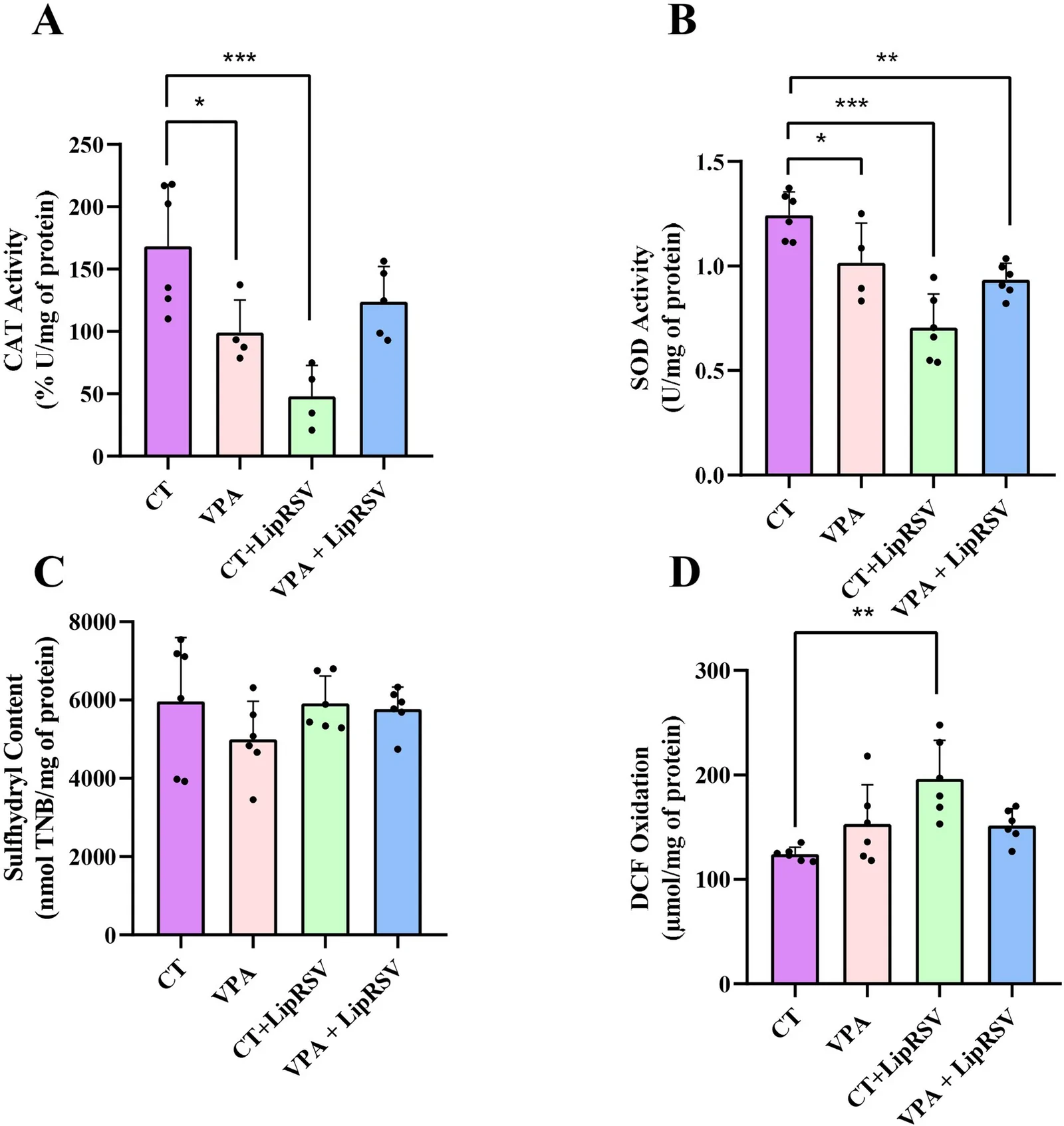
Evaluation of oxidative stress parameters in the Frontal Cortex. Effects of prenatal exposure to VPA and postnatal treatment with LipRSV on (**A**) CAT activity; (**B**) SOD activity; (**C**) sulfhydryl content; and (**D**) DCF oxidation levels. Data are expressed as mean ± standard deviation (SD). Statistical analysis was performed using one-way ANOVA followed by Tukey’s post hoc test for multiple group comparisons. For pairwise comparisons between the CT and VPA group, Student’s T-test was used. *p < 0.05, **p < 0.01, ***p < 0.001. (*n* = 3–5 animals/group)

In the activity of the catalase enzyme (Fig. 6A), a significant reduction was observed in the VPA group (*p < 0.05) and the CT+LipRSV group (***p < 0.001) when compared to the CT group. Regarding SOD activity (Fig. 6B), a significant decrease was observed in the VPA group (*p < 0.05), CT+LipRSV group (****p < 0.0001), and in the VPA+LipRSV group (**p < 0.01) compared to the CT group. The CT+LipRSV group also showed a significant reduction (*p < 0.05) compared to the VPA group. No significant differences were found in the sulfhydryl content (Fig. 6C). In contrast, DCF oxidation (Fig. 6D) showed a significant increase (**p < 0.01) in the CT+LipRSV compared to the CT group.

#### Hypothalamus

**Fig. 7:**
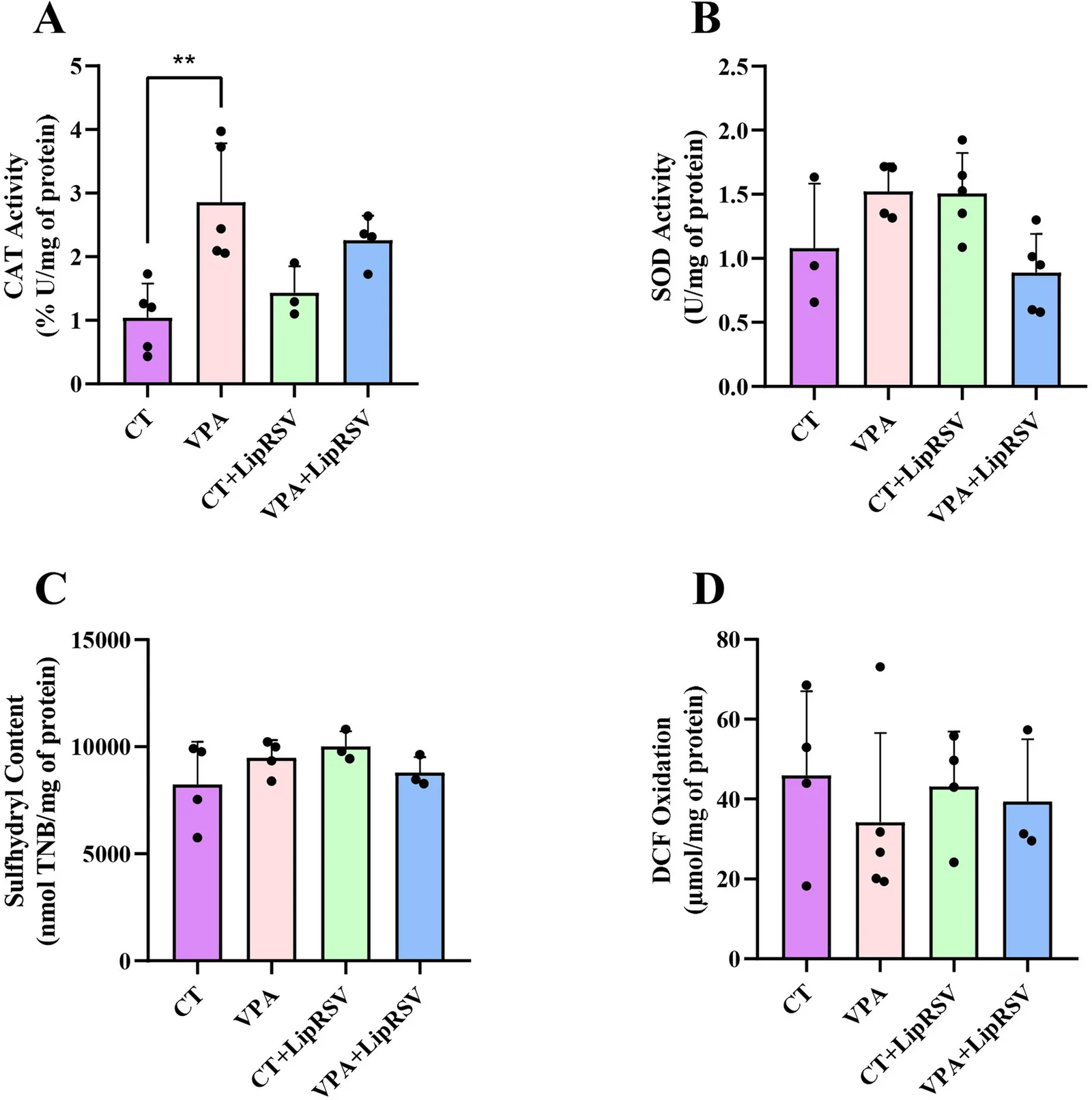
Evaluation of oxidative stress parameters in the Hypothalamus. Effects of prenatal exposure to VPA and postnatal treatment with LipRSV on (**A**) CAT activity; (**B**) SOD activity; (**C**) sulfhydryl content; and (**D**) DCF oxidation levels. Data are expressed as mean ± standard deviation (SD). Statistical analysis was performed using one-way ANOVA followed by Tukey’s post hoc test for multiple group comparisons. For pairwise comparisons between the CT and VPA groups, Student’s t-test was used. *p < 0.05, **p < 0.01. (*n* = 3–5 animals/group)

In the catalase activity assay (Fig. 7A), the VPA group showed a significant increase (**p < 0.01) in comparison to the CT group. No significant differences were observed among the groups for SOD activity, sulfhydryl content and DCF oxidation assays (Fig. 7B, C and D, respectively).

#### Hippocampus

**Fig. 8:**
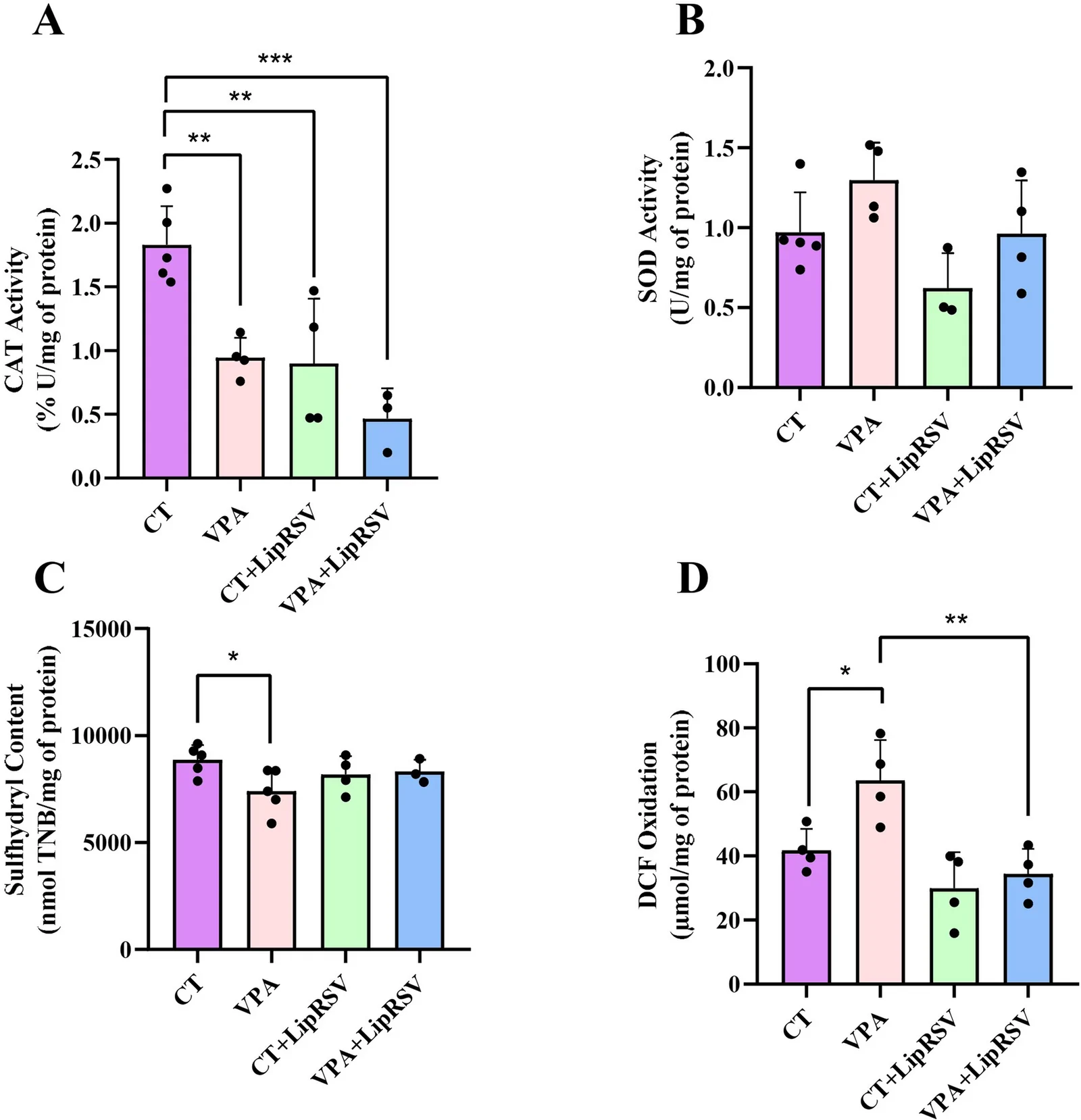
Evaluation of oxidative stress parameters in Hippocampus. Effects of prenatal exposure to VPA and postnatal treatment with LipRSV on (**A**) CAT activity; (**B**) SOD activity; (**C**) sulfhydryl content; and (**D**) DCF oxidation levels. Data are expressed as mean ± standard deviation (SD). Statistical analysis was performed using one-way ANOVA followed by Tukey’s post hoc test for multiple group comparisons. For pairwise comparisons between the CT and VPA groups, Student’s t-test was used. *p < 0.05, **p < 0.01, ***p < 0.001. (*n* = 3–5 animals/group)

Regarding catalase activity (Fig. 8A), the VPA and CT+LipRSV groups exhibited a significant reduction (**p < 0.01), while the VPA+LipRSV group showed an even more pronounced reduction (***p < 0.001) compared to the CT group. No significant differences were observed among the groups for SOD activity assay (Fig. 8B). For sulfhydryl content (Fig. 8C), the VPA group displayed a significant reduction (*p < 0.05) in comparison to the CT group. In the DCF oxidation assay (Fig. 8D), the VPA group exhibited a significant increase (*p < 0.05) compared to the CT group, whereas VPA+LipRSV groups showed significant reductions (**p < 0.01) compared to the VPA group.

#### Striatum

**Fig. 9:**
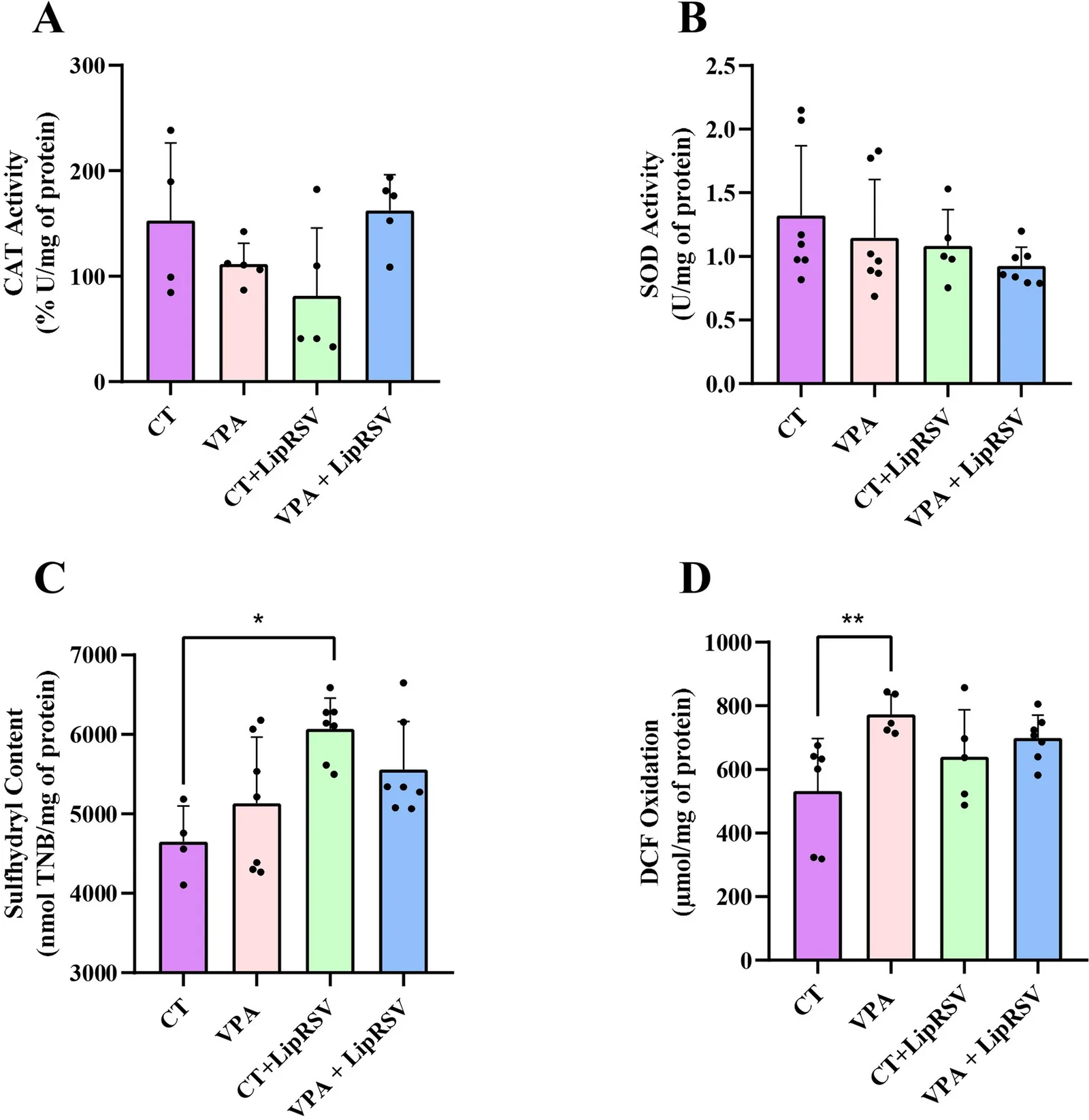
Evaluation of oxidative stress parameters in the Striatum. Effects of prenatal exposure to VPA and postnatal treatment with LipRSV on (**A**) CAT activity; (**B**) SOD activity; (**C**) sulfhydryl content; and (**D**) DCF oxidation levels. Data are expressed as mean ± standard deviation (SD). Statistical analysis was performed using one-way ANOVA followed by Tukey’s post hoc test for multiple group comparisons when data followed a normal distribution. For non-normally distributed data, the Kruskal–Wallis test was applied followed by Dunn’s post hoc test. Pairwise comparisons between CT and VPA groups were performed using the Mann–Whitney U test. *p < 0.05, **p < 0.01. (*n* = 3–5 animals/group)

In the catalase and SOD activities (Fig. 9A and B, respectively), no significant differences were observed between the groups. In the sulfhydryl content (Fig. 9C), only the CT+LipRSV group showed a significant increase (*p < 0.05) compared to the CT group. Regarding DCF oxidation (Fig. 9D), the VPA group showed a significant increase (**p < 0.01) compared to the CT group.

#### Posterior Cortex

**Fig. 10:**
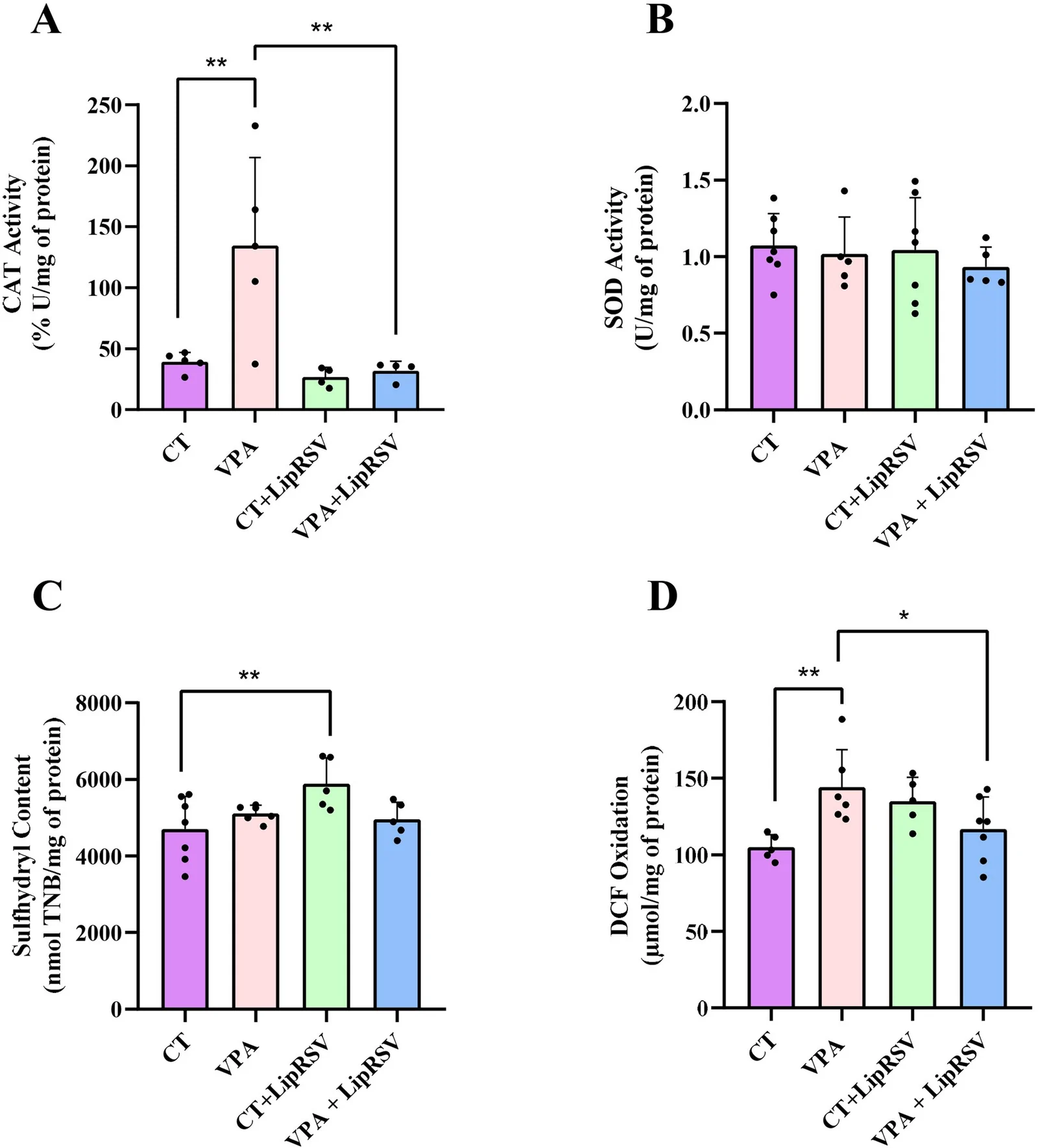
Evaluation of oxidative stress parameters in the Posterior Cortex. Effects of prenatal exposure to VPA and postnatal treatment with LipRSV on (**A**) CAT activity; (**B**) SOD activity; (**C**) sulfhydryl content; and (**D**) DCF oxidation levels. Data are expressed as mean ± standard deviation (SD). Statistical analysis was performed using one-way ANOVA followed by Tukey’s post hoc test for multiple group comparisons. For non-normally distributed data, pairwise comparisons between the CT and VPA groups were performed using the Mann–Whitney U test. *p < 0.05, **p < 0.01. (*n* = 3–5 animals/group)

In catalase activity (Fig. 10A), a significant increase (**p < 0.01) was observed in the VPA group compared to the CT group, while the VPA+LipRSV group showed a significant decrease (**p < 0.01) compared to the VPA group. No significant changes were observed in SOD activity (Fig. 10B). Regarding sulfhydryl content (Fig. 10C), a significant increase was found in the CT+LipRSV group (**p < 0.01) compared to the CT group. In DCF oxidation (Fig. 10D), the VPA group exhibited a significant increase (**p < 0.01) compared to CT, whereas the VPA+LipRSV group showed reduced DCF oxidation levels compared to the VPA group (*p < 0.05).

#### Cerebellum

**Fig. 11:**
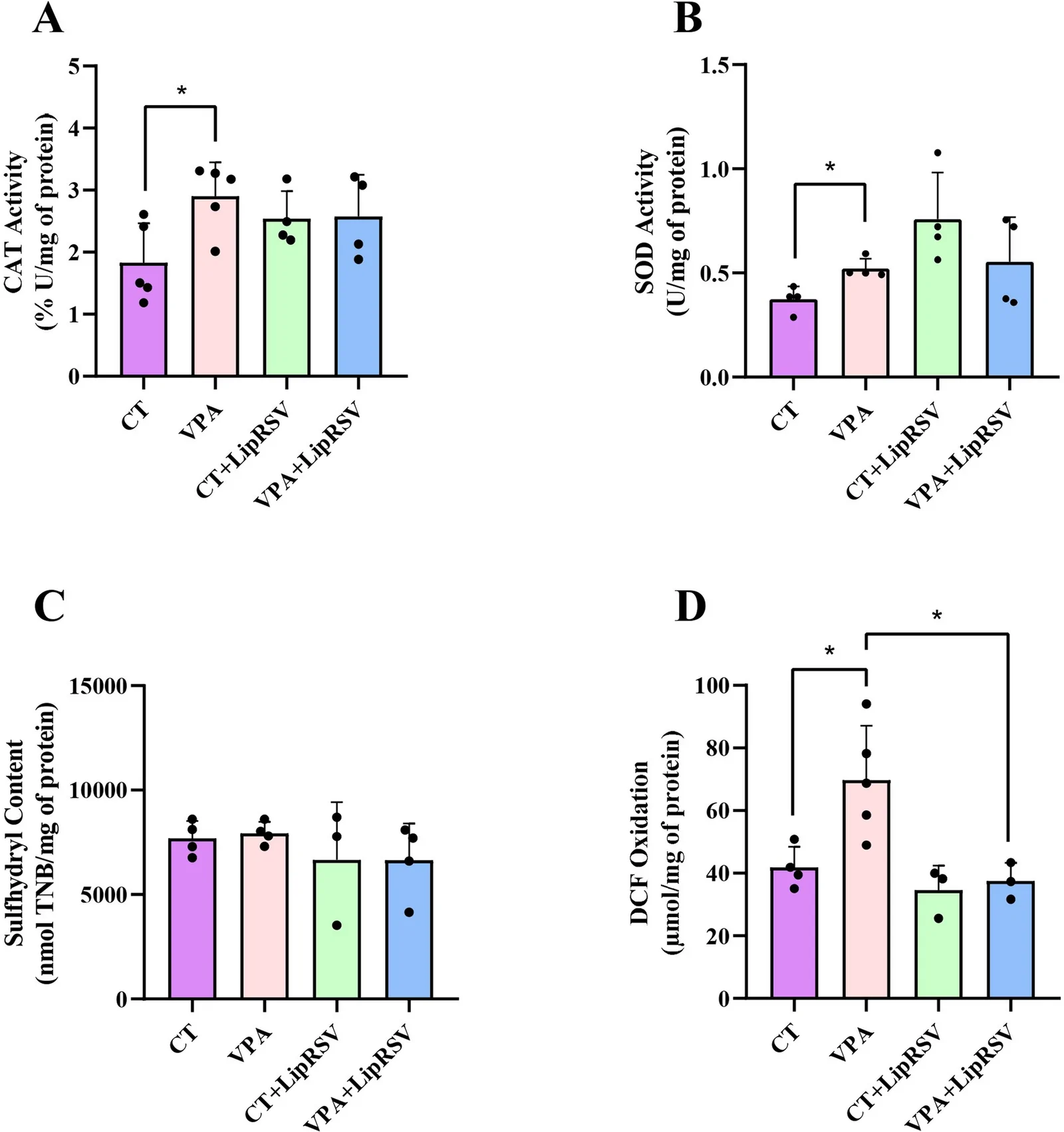
Evaluation of oxidative stress parameters in the Cerebellum. Effects of prenatal exposure to VPA and postnatal treatment with LipRSV on (**A**) CAT activity; (**B**) SOD activity; (**C**) sulfhydryl content; and (**D**) DCF oxidation levels. Data are expressed as mean ± standard deviation (SD). Statistical analysis was performed using one-way ANOVA followed by Tukey’s post hoc test for multiple group comparisons. For non-normally distributed data, pairwise comparisons between the CT and VPA groups were performed using the Mann–Whitney U test. *p < 0.05, **p < 0.01. (*n* = 3–5 animals/group)

Regarding catalase activity (Fig. 11A), the VPA group exhibited a significant increase (*p < 0.05) in CAT activity compared to the CT group. In SOD activity (Fig. 11B), an increase was observed (*p < 0.05) in the VPA and the CT+LipRSV compared to the CT group. No significant differences were observed among groups in sulfhydryl content (Fig. 11C). In the DCF oxidation assay (Fig. 11D), the VPA group presented a significant increase (*p < 0.05) compared to the CT group. Besides, VPA+LipRSV demonstrated significant reduction in DCF oxidation levels (*p < 0.05, respectively) when compared to the VPA group.

Given the heterogeneous pattern of oxidative stress markers across brain regions, the main results were organized in Table 2. In addition to summarizing individual parameters, an integrated redox profile was assigned to each brain region analyzed. This classification was based on the premise that oxidative stress reflects an imbalance between increased oxidative damage and reduced antioxidant capacity. Thus, a region was classified as presenting “oxidative stress” (OS) when DCF was increased together with a reduction in at least one antioxidant defense parameter, such as sulfhydryl content, CAT or SOD activity.

**Table 2:** Summary of the evaluation of the redox state of the cerebral homogenized of males-overview

		VPA	CT+LipRSV	VPA+LipRSV
Frontal Cortex	CAT	↓	↓↓↓	-
	SOD	**↓**	**↓↓↓**	**↓↓**
	Sulfhydryl	-	-	-
	DCF	-	**↑↑**	-
	OS	0	✓	0
Hypothalamus	CAT	**↑↑**	-	-
	SOD	-	-	-
	Sulfhydryl	-	-	-
	DCF	-	-	-
	OS	0	0	0
Hippocampus	CAT	**↓↓**	**↓↓**	**↓↓↓**
	SOD	-	-	-
	Sulfhydryl	**↓**	-	-
	DCF	**↑**	-	↓↓*
	OS	✓	0	0
Striatum	CAT	-	-	-
	SOD	-	-	-
	Sulfhydryl	-	**↑**	-
	DCF	**↑↑**	-	-
	OS	✓	0	0
Posterior Cortex	CAT	**↑↑**	-	↓↓*
	SOD	-	-	-
	Sulfhydryl	-	**↑↑**	-
	DCF	**↑↑**	-	↓*
	OS	✓	0	0
Cerebellum	CAT	**↑**	-	-
	SOD	**↑**	-	-
	Sulfhydryl	-	-	-
	DCF	**↑**	-	↓*
	OS	✓	0	0

CAT activity, catalase enzyme ativity; SOD, superoxide dismutase activity; Sulfhydryl, sulfhydryl content; DCF, 2’7’ - dichlorofluorescein oxidation; OS, oxidative stress

↑ - Represents an increase in the evaluated parameter (*p* < 0.05)

↑↑ - Represents an increase in the evaluated parameter (*p* < 0.01)

↑↑↑ Represents an increase in the evaluated parameter (*p* < 0.001)

↓ - Represents an decrease in the evaluated parameter (*p* < 0.05)

↓↓ - Represents an decrease in the evaluated parameter (*p* < 0.01)

↓↓↓ - Represents an decrease in the evaluated parameter (*p* < 0.001)

- There is no statistical difference between the groups evaluated

✓ There was oxidative stress

0 There was not oxidative stress

Arrows is compared to CT group

Arrows with * is compared with VPA group

### Cytokines Levels in Brain and Plasma of Male Rats

Besides brain analysis, 1 mL of blood was collected from each animal to assess systemic neuroinflammatory markers. The levels of tumor necrosis factor (TNF-α), brain-derived neurotrophic factor (BDNF), and nerve growth factor (NGF) were quantified in plasma samples to determine whether LipRSV modulated peripheral inflammatory responses in the VPA-induced autism model.

#### Immunoenzymatic Analysis of Posterior Cortex Content

**Fig. 12:**
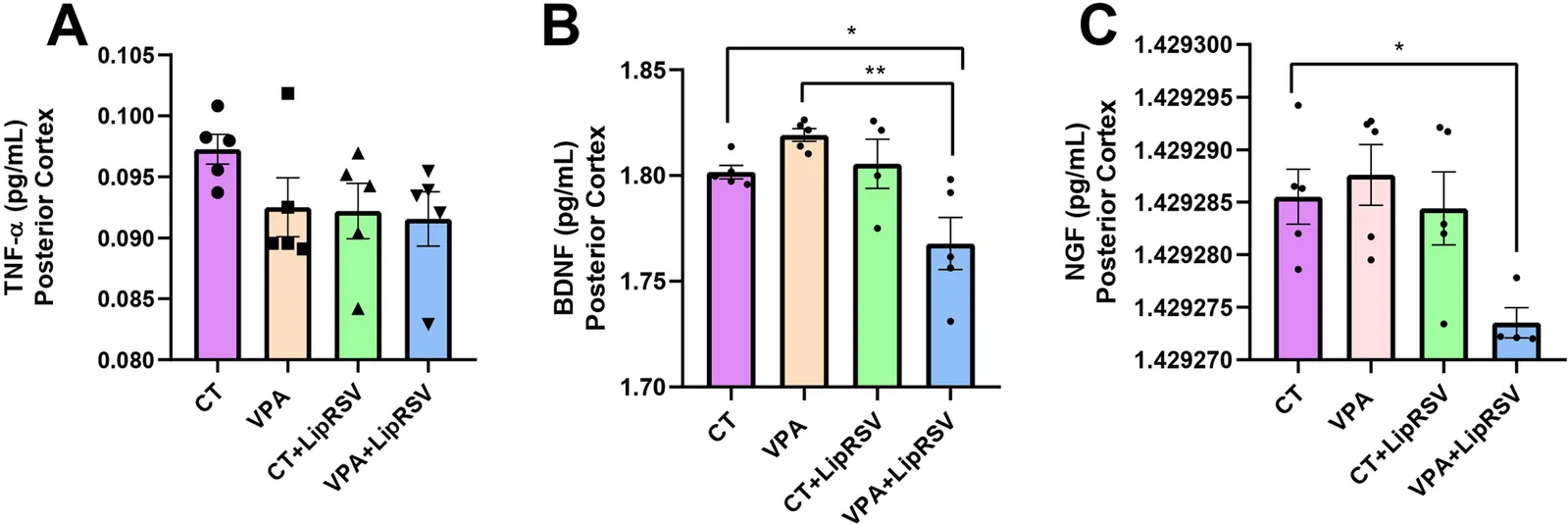
Concentrations of TNF-α, BDNF and NGF in the posterior cortex of animals in the VPA-induced autism model treated with LipRSV. Effects of prenatal exposure to VPA and postnatal treatment with LipRSV on (**A**) TNF-α, (**B**) BDNF, and (**C**) NGF levels in the posterior cortex. The experimental groups included saline-treated control animals (CT), VPA-exposed animals (VPA), animals treated with saline and liposomes containing resveratrol (CT+LipRSV), and VPA-exposed animals treated with LipRSV (VPA+LipRSV). Concentrations of TNF-α, BDNF, and NGF were determined using specific ELISA assays. Data are expressed as mean ± standard deviation (SD). Statistical analysis was performed using one-way ANOVA followed by Tukey’s post hoc test. *p < 0.05; **p < 0.01 vs. VPA. (*n* = 3–5 animals/group)

TNF-α levels (Fig. 12A) showed no statistically significant differences among the groups (CT, VPA, CT+LipRSV, and VPA+LipRSV). In contrast, there was a significant reduction in BDNF levels (Fig. 12B) in the VPA+LipRSV group compared to the CT group (*p < 0.05) and the VPA group (**p < 0.01). Regarding NGF levels, the VPA + LipRSV group presented lower levels (*p < 0.05) in comparison to the VPA group (Fig. 12C).

## Discussion

This study demonstrates that PEGylated liposomal RSV reduces oxidative stress in a region-specific manner in a VPA-induced model of autism, although these biochemical effects were heterogeneous across brain regions. However, despite these improvements in redox balance, no significant recovery of behavioral deficits was observed, suggesting a heterogeneous relationship between molecular and functional outcomes. In addition, treatment did not restore neurotrophin signaling and was associated with reductions in BDNF and NGF levels, suggesting a limited and potentially unfavorable impact on pathways involved in synaptic plasticity and neurodevelopment. Notably, while PEGylated liposomes showed good hemocompatibility, the inclusion of phosphatidylcholine increased hemolytic activity, which was mitigated by RSV incorporation. Together, these findings demonstrate a clear heterogeneity between the modulation of oxidative stress and the rescue of behavioral deficits in a developmental model of autism. This finding challenges the assumption that targeting oxidative stress alone is sufficient to ameliorate ASD-like phenotypes, particularly when interventions are applied after critical neurodevelopmental windows.

Biotechnology has advanced drug delivery through nanoparticle systems (Gaucher et al. 2005; Peer et al. 2007). Conventional drugs show limitations in neurological disorders, especially brain tumors, as oral administration leads to hepatic metabolism and reduced concentration at the target (Tang et al. 2004). Their nonspecific distribution also causes toxicity in organs such as liver, kidneys, lungs, and heart (van Erven and Schalij 2010). Amphiphilic macromolecules are attractive carriers since lipids form vesicles like biological membranes (Lasic and Papahadjopoulos 1995). These structures present low toxicity and allow incorporation of lipophilic, amphiphilic, and hydrophilic compounds (Ali et al. 2013; Daneshpour et al. 2011), enabling controlled circulation times, reduced side effects, and optimized therapeutic action (Umrethia et al. 2007).

LipRSV formulations were prepared according to parameters defined by dynamic light scattering (DLS), which measured polydispersity index (PDI), hydrodynamic radius (RH), and zeta potential (PZ) (de Castro et al. 2019; dos Santos et al. 2018; Cardoso dos Santos et al. 2021). Although nanoparticles of 100–300 nm are optimal for brain delivery (Hong et al. 2019), liposomes above this range may still cross the BBB (Salama et al., 2012). Although some studies suggest that PEGylated liposomes may facilitate CNS delivery, the physicochemical characteristics observed in the present study (343 nm; zeta potential − 51.3 mV) are not considered optimal for BBB permeability according to current nanoneuroscience literature (Hong et al. 2019). Therefore, direct CNS delivery cannot be assumed from the present data. PEG incorporation extends circulation, reduces uptake by liver and spleen, and shows no toxicity (Gregoriadis 1995; Li et al. 2017). Liposomes were synthesized using PC and PC + C22PEG900GlcNAc, with RSV encapsulated in both.

RSV’s low bioavailability compromises its benefits, but encapsulation may overcome these limitations (Neves et al. 2012). Since toxicity and activity depend on physicochemical traits, size, zeta potential, and morphology were evaluated (Cardoso dos Santos et al. 2021). LipRSV exhibited a strongly negative zeta potential (–51.3 mV), conferring high stability but possibly limiting BBB interactions. Drug encapsulation increased particle size by 21.7 nm, consistent with RSV insertion. Transmission electron microscopy could further confirm efficiency.

Interestingly, (Salegio et al. 2014) demonstrated that nanoparticles infused into the brain can be transported to distal sites in a manner that depends on both the physicochemical properties of the carrier system and the target brain region. The distribution mechanisms varied according to the neural territory and particle type, resulting in distinct regional patterns of nanoparticle distribution and retention. Likewise, (Rabanel et al. 2020) reported that, although parameters such as particle size and PEGylation influence nanoparticle cellular uptake, these characteristics exert only a limited effect on their overall translocation to the central nervous system. These findings suggest that, even when key physicochemical properties are optimized, uniform brain distribution is not guaranteed, potentially contributing to region-specific therapeutic responses.

Hemocompatibility tests were conducted to evaluate safety. Nanoparticles can cross membranes, accumulate in organs, and potentially cause tissue damage (Nel et al. 2006). In blood, they interact mainly with erythrocytes, inducing alterations in morphology and function, sometimes leading to hemolysis (Pan et al. 2016; Tsui et al. 2020). Our results showed general safety of LipRSV, except for mixed liposomes, which caused hemolysis. Similar findings were reported for melphalan and methotrexate-loaded liposomes, where hemolysis was absent despite other immune effects (Kuznetsova et al. 2012). Thus, hemoreactivity may depend more on drug fraction than liposome structure. Confirmed safety allowed progression to in vivo testing for ASD models. Interestingly, while PEGylated liposomes alone exhibited good hemocompatibility and low toxicity, the combination of phosphatidylcholine and PEG resulted in increased hemolytic activity, which was mitigated by the addition of RSV. This suggests a dual role of RSV not only as an antioxidant agent but also as a stabilizing component within the liposomal formulation. However, the possibility that, under certain conditions, RSV may exert pro-oxidant effects should not be overlooked. The balance between antioxidant and pro-oxidant activity is known to depend on factors such as concentration, cellular environment, and redox state, and may partially explain the variability in biological outcomes.

Autism is a complex neurodevelopmental disorder with increasing diagnoses (Hyman et al. 2020; Zyoud et al. 2023). Animal models, such as prenatal VPA exposure, replicate human symptoms and allow mechanistic studies (Schneider and Przewłocki 2005; Sharma et al. 2024). In our study, pups were monitored for weight, motor, and behavioral development. VPA reduced body weight, confirming previous reports (Du et al. 2017; Schneider and Przewłocki 2005). Interestingly, LipRSV alone also reduced pup weight, suggesting metabolic effects of RSV, possibly through energy regulation and mitochondrial biogenesis (Price et al. 2012).

In olfactory discrimination, VPA and group showed higher latency to find the nest, indicating orientation impairments. LipRSV did not fully restore performance, although other studies suggest RSV can improve ASD-like behaviors (Zambrelli et al. 2021). Negative geotaxis showed no significant differences, consistent with variability described by (Motz and Alberts 2005). Exploratory activity was assessed in the open field. Both CT and VPA groups showed decreased rearing and crossings on day 2, reflecting habituation, but no significant group differences emerged (Singh 2020). These findings indicate that LipRSV treatment was insufficient to promote detectable behavioral improvements under the experimental conditions used.Social behavior was impaired in VPA and CT+LipRSV groups, with increased latency to interaction and reduced total interaction time. Both also spent more time in the empty chamber, indicating social aversion. These deficits are consistent with VPA literature (Roullet et al. 2013)and suggest LipRSV treatment did not reverse them. RSV efficacy may depend on formulation, dose, or bioavailability (Malaguarnera et al. 2020). Importantly, the observation that CT+LipRSV animals also displayed impaired social interaction parameters suggests that the formulation itself may influence behavioral responses independently of prenatal VPA exposure.

ASD is a neurodevelopmental disorder with still poorly understood pathophysiology and multifactorial etiology. However, it is known that individuals with ASD are more prone to oxidative stress and consequently exhibit altered levels of antioxidant enzymes and compounds (Bjørklund et al. 2020).

The oxidation of DCFH-DA into DCF is widely used to quantify total ROS in cells, especially hydroxyl radicals. This process is detected through the fluorescence emitted by DCF in response to the oxidation of DCFH by ROS (Kim and Xue 2020). In the present study, DCFH fluorescence levels were consistently higher in the VPA group compared to the CT group in the cerebellum, hippocampus, posterior cortex, and striatum, indicating increased oxidative stress and supporting the effectiveness of the VPA-induced animal model. These findings were also reported by (Campos et al. 2025) in a VPA-induced animal model of autism. In the posterior cortex, a reduction in DCFH levels was observed in the VPA+LipRSV group compared to the VPA group, suggesting that LipRSV treatment partially attenuated ROS production in this region. In contrast, in the frontal cortex, the CT+LipRSV group showed an increase in ROS formation compared to the CT group.

Interestingly, in the frontal cortex, LipRSV treatment in CT animals was associated with reduced antioxidant enzyme activity together with increased ROS formation, suggesting a potential pro-oxidant or redox-disruptive effect in this brain region. These findings contrast with the expected antioxidant role of LipRSV and reinforce that its biological effects may vary according to the brain region and local redox environment. The heterogeneous oxidative profile observed across brain regions likely reflects intrinsic metabolic and cellular differences among neural structures. Regions such as the hippocampus and cerebellum are particularly vulnerable to ROS accumulation due to high mitochondrial activity and synaptic demand, whereas frontal cortical areas may exhibit distinct redox buffering capacities. Additionally, RSV is known to exert context-dependent antioxidant or pro-oxidant effects depending on concentration, transition metal availability, mitochondrial state, and local enzymatic activity. RSV may exhibit biphasic redox behavior, acting as an antioxidant under oxidative stress conditions but inducing pro-oxidant effects in physiological environments or at specific concentrations (de la Lastra and Villegas 2007; Plauth et al. 2016; Shaito et al. 2020). These factors may partially explain the paradoxical increase in DCF oxidation observed in the frontal cortex of CT+LipRSV animals.

As part of the assessment of oxidative damage, sulfhydryl group quantification was performed. Sulfhydryl groups are considered sensitive biomarkers of oxidative stress due to their central role in the cellular antioxidant system. Significant reductions in these groups indicate increased oxidative damage and reduced antioxidant capacity (Pérez et al. 2012). In the present study, a decrease in sulfhydryl levels was observed in the hippocampus of the VPA group compared to the CT group, suggesting elevated oxidative stress in this region. In contrast, increased sulfhydryl levels were found in the posterior cortex and striatum of the CT+LipRSV group relative to the CT, indicating a possible positive modulation of the redox system by LipRSV treatment. Together, these findings support the hypothesis that VPA induces oxidative stress in specific brain regions, such as the hippocampus, while LipRSV may exert region-specific antioxidant effects, though not uniformly across the brain.

The results obtained for CAT activity indicate a heterogeneous modulation of the antioxidant system across different brain regions in response to VPA exposure and LipRSV treatment. CAT activity was reduced in the frontal cortex in the VPA and CT+LipRSV groups, and a decrease was observed in the hippocampus across all experimental groups (VPA, CT+LipRSV, and VPA+LipRSV). Conversely, increased CAT was observed in the hypothalamus and posterior cortex of VPA-exposed animals. However, in the posterior cortex, CAT activity was reduced in the VPA+LipRSV group compared to the VPA group, indicating that LipRSV treatment may have negatively modulated the enzyme’s expression or activity, either by attenuating oxidative stress and thus reducing enzymatic defense needs, or through a possible pro-oxidant effect dependent on the region and experimental conditions. These findings highlight that the effects of VPA and LipRSV on the redox system vary regionally and may be influenced by local factors such as the specific metabolism of each brain structure.

Another important point of the present findings is the region-specific modulation of oxidative stress. The differential effects observed across brain regions suggest that liposomal deliverymay reflect differences in regional susceptibility or indirect systemic effects rather than confirmed differential brain distribution. Factors such as regional BBB permeability, local metabolic demands, and microenvironmental conditions may influence the uptake and efficacy of PEGylated liposomes. This spatial heterogeneity may contribute to the partial biochemical response observed and further explain the lack of global behavioral improvement. The region-specific redox modulation observed here underscores the heterogeneity of oxidative vulnerability in the developing brain. While LipRSV reduced oxidative markers in the hippocampus, cerebellum and posterior cortex, the increase observed in the frontal cortex suggests that nanocarrier distribution and local redox balance may critically influence therapeutic outcomes.

Besides, it seems that behavioral phenotypes in the VPA model emerge from altered developmental trajectories rather than acute oxidative imbalance, which may explain why postnatal redox modulation failed to rescue sociability and exploratory behavior. Furthermore, it is important to note that the dissociation between biochemical improvements and behavioral outcomes remains a significant challenge in the neurobiology of ASD, and the findings of the present study fit coherently within this context. A recent clinical trial evaluating glutathione, vitamin C, and N-acetylcysteine reported no significant differences between treatment and placebo groups in either biochemical or behavioral measures, with the modest improvements observed being attributed to nonspecific effects related to clinical follow-up and study routine (Williams et al. 2025). In turn, (Ornoy et al. 2019), in their review of antioxidant interventions in animal models of ASD, reported that these strategies frequently reduce oxidative stress and improve certain behavioral parameters, although many of these effects appear to depend on the continuous administration of the treatment. Collectively, these findings suggest that behavioral recovery in ASD models involves mechanisms that are more complex than the mere normalization of oxidative stress markers, requiring the integration of multiple neural circuits and pathophysiological processes.

CAT and SOD activities have been widely studied in VPA-induced autism models, but antioxidant responses appear to vary according to tissue, brain region, developmental stage, and experimental conditions. A previous study from our group did not observe significant changes in CAT activity in the hippocampus and cortex following VPA exposure, reinforcing the variability of antioxidant responses in CNS tissues (Costa et al., 2025). In contrast, Bambini-Junior et al. (2011) reported alterations in liver parameters in the VPA-induced autism model, suggesting that VPA-induced redox imbalance may also involve peripheral organs. However, these peripheral findings should not be interpreted as direct evidence of brain-specific CAT modulation. Together, these findings highlight the need for further studies investigating how peripheral and central alterations interact in VPA-induced neurodevelopmental impairment (Bambini-Junior et al. 2011). The analysis of SOD activity revealed variable responses depending on the brain region and treatment applied. In the frontal cortex, the reduction of SOD observed in the VPA, CT+LipRSV and VPA+LipRSV groups compared to CT suggests a decreased antioxidant defense in this region, potentially increasing vulnerability to oxidative stress. Conversely, the increased SOD activity in the cerebellum of the VPA group suggests compensatory antioxidant responses in specific regions. Therefore, these results demonstrate that both VPA exposure and LipRSV treatment provoke distinct regional alterations in the antioxidant system. Additionally, prenatally VPA-treated rats showed higher levels of malondialdehyde (MDA), a marker of oxidative stress, and lower levels of glutathione peroxidase (GPX) and SOD (Farbin et al. 2024), indicating an imbalance in the antioxidant system. These findings support our results of reduced SOD activity and variable CAT activity in different brain regions, suggesting that VPA exposure impairs antioxidant capacity and may increase oxidative damage. Although LipRSV treatment has not been widely studied in animal models of autism, one study showed that RSV treatment prevented changes in neuronal organization and the number of GABAergic interneurons in the frontal cortex and hippocampus of VPA-exposed rats, suggesting that RSV may have protective effects in specific brain regions (Santos-Terra et al. 2022).

The regional heterogeneity of oxidative stress in the brains of individuals with ASD is a phenomenon consistently documented in literature. (Sajdel-Sulkowska et al. 2011)were the first to demonstrate region-specific alterations in the oxidative damage marker 3-nitrotyrosine (3-NT) in postmortem brains of individuals with autism, showing that regions such as the orbitofrontal cortex, Wernicke’s area, the cerebellar vermis, and the cerebellar hemispheres exhibited distinct levels of this biomarker, reflecting region-specific redox signatures. Supporting the existence of regional redox alterations in ASD, (Rose et al. 2012) evaluated oxidative stress markers in the cerebellum and temporal cortex (BA22) and observed reductions in GSH levels and the GSH/GSSG ratio, along with increased 3-NT and 8-oxo-dG levels in both regions. Although pro-oxidant profile was evident in both brain areas, differences in the magnitude of some alterations and in aconitase activity suggested that the functional consequences of oxidative stress may vary across brain regions. These findings provide support for the interpretation of the present results, in which different brain regions exhibited distinct responses to LipRSV treatment, reflecting the intrinsic redox heterogeneity of the autistic brain rather than an inconsistency of the experimental model.

**Fig. 13:**
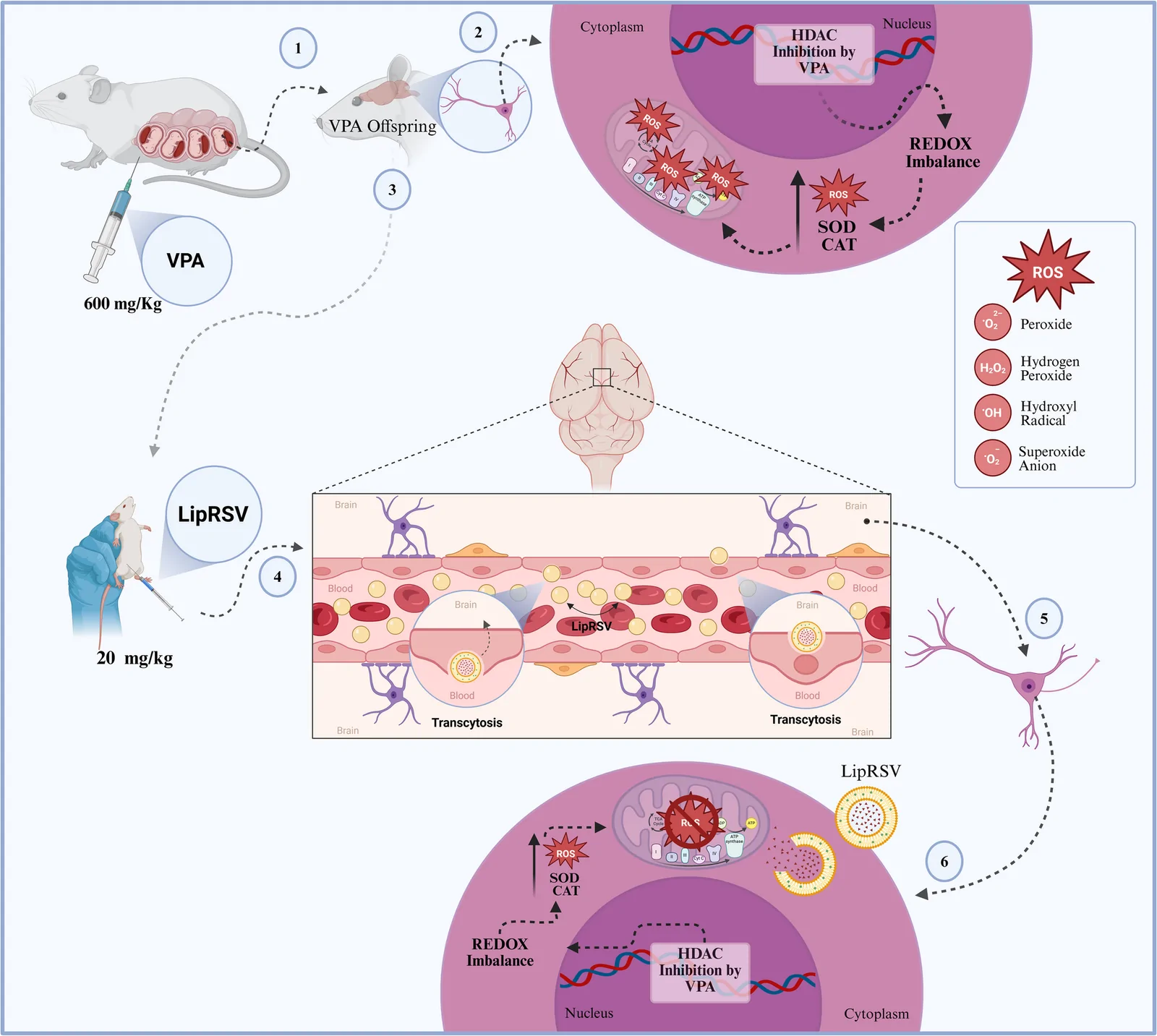
Proposed conceptual framework of LipRSV effects in the VPA-induced autism model. Prenatal exposure of male Wistar rats to valproic acid (VPA, 600 mg/kg) inhibits HDACs, leading to redox imbalance characterized by increased production of reactive oxygen species (ROS) and compensatory modulation of antioxidant enzymes. Postnatal treatment with liposomes composed of phosphatidylcholine and polyethylene glycol containing resveratrol (LipRSV, 3.33 mg/kg) may indirectly modulate oxidative stress pathways associated with prenatal VPA exposure. Direct BBB penetration was not evaluated in the present study. The proposed model illustrates region-specific and potentially bidirectional redox responses induced by LipRSV treatment, including antioxidant effects in some brain regions and possible pro-oxidant effects under specific local metabolic conditions

Figure 13 illustrates the effects of prenatal exposure to VPA, showing that HDAC inhibition may exacerbate oxidative stress by dysregulating the expression of genes involved in the antioxidant response, thereby promoting redox imbalance through increased ROS production and impaired antioxidant defenses (Gottlicher 2001; Phiel et al. 2001) treatment with LipRSV may counteract these alterations.

In addition to oxidative stress, the inflammatory process is also associated with the pathophysiology of ASD. Studies show that individuals with ASD exhibit low-grade inflammation in both the central nervous system and peripheral tissues. This condition may influence neurodevelopment and contribute to behavioral changes related to the disorder (Alzghoul et al. 2019; Jiang et al. 2018). In this sense, TNF-α, NGF, and BDNF are essential molecules for the proper functioning of the nervous system. Each plays specific roles in neuronal development and maintenance, and changes in their levels or signaling pathways may be involved in various neuropathologies, including ASD. Neuroinflammation induced by TNF-α can affect the signaling of neurotrophins such as NGF and BDNF, which are essential for neuronal survival, growth, and plasticity (Saghazadeh and Rezaei 2017).

Immunoenzymatic analysis revealed no statistically significant differences in TNF-α levels in the posterior cortex among the experimental groups. However, BDNF levels were significantly reduced in the VPA+LipRSV group compared to both the CT and VPA groups, indicating a negative effect of LipRSV treatment on the expression of this neurotrophin. NGF levels were also significantly lower in the VPA+LipRSV group compared to the CT group, suggesting that LipRSV treatment may have contributed to a further reduction in NGF levels. The lack of normalization of BDNF and NGF levels suggests that LipRSV did not engage plasticity-related pathways essential for behavioral rescue, reinforcing the notion that antioxidant strategies alone may be insufficient in ASD models.

Despite the observed reductions in oxidative stress, LipRSV treatment did not restore the BDNF alterations induced by prenatal VPA exposure, reinforcing the notion that redox modulation alone is insufficient to normalize neurobiological pathways associated with ASD. LipRSV was linked to a further decrease of BDNF and NGF in the posterior cortex. Neurotrophins such as BDNF and NGF play central roles in synaptic plasticity, neuronal survival, and network organization, and their dysregulation may represent a more proximal mechanism underlying behavioral impairments. These results show that LipRSV has limited impact on VPA-induced neurochemical changes under the tested conditions. Additional studies are needed to clarify the mechanisms involved and to improve LipRSV treatment approaches.

Some limitations of this study should be acknowledged. First, the biodistribution of the liposomal formulation was not directly assessed, limiting the ability to correlate regional biochemical effects with drug delivery efficiency. In addition, the encapsulation efficiency of RSV in PEGylated liposomes was not determined. Therefore, the exact proportion of encapsulated versus non-encapsulated RSV, as well as the actual encapsulated RSV dose administered in vivo, could not be established. Although physicochemical characterization demonstrated appropriate vesicle size distribution, polydispersity index, zeta potential, and formulation stability, and the increase in vesicle size after RSV incorporation supported the association of RSV with the liposomal system, these parameters do not replace direct quantification of encapsulation efficiency. Second, only a single treatment window was evaluated, precluding conclusions about the impact of earlier or prolonged interventions. Third, the present study used only male offspring. Male rodents are frequently used in the VPA-induced ASD model because male animals often display more consistent behavioral alterations in this model (Kazlauskas et al., 2019; Longo et al., 2025). Nevertheless, sex is an important biological variable in ASD and may influence neurodevelopmental trajectories, behavioral outcomes, oxidative stress responses, and treatment effects. Therefore, the present findings should not be generalized to females. Future studies including both male and female offspring, with adequate power to evaluate sex-by-treatment interactions, are needed to determine whether LipRSV produces sex-specific behavioral and biochemical effects. Finally, behavioral assessments were limited to specific domains and the observed biochemical changes cannot be interpreted as evidence of global neuroprotection, since behavioral deficits persisted and some molecular markers were negatively affected by treatment. Future studies should include a broader assessment of functional outcomes and explore sex-specific responses to LipRSV treatment in both male and female animals. In addition, different therapeutic windows, treatment durations, and dosing regimens should be investigated to better clarify the influence of sex and developmental timing on treatment efficacy in ASD models.

## Conclusion

In conclusion, PEGylated liposomal RSV induced partial and region-specific modulation of oxidative stress markers in a developmental VPA model of ASD. Favorable reductions in ROS levels were restricted to the hippocampus, posterior cortex, and cerebellum, whereas other brain regions showed heterogeneous or unfavorable redox responses, including alterations in antioxidant enzyme activity. Importantly, these biochemical changes were not accompanied by behavioral recovery. In addition, LipRSV treatment was associated with reduced BDNF and NGF levels, further indicating that redox modulation was not paralleled by neurotrophin restoration.Together, these results suggest limited therapeutic benefit of the formulation under the experimental conditions tested and highlight the complexity of linking redox modulation to functional behavioral outcomes in ASD models. Future studies should optimize treatment parameters, including formulation, dosage, timing of administration, and assessment of brain biodistribution and explore multi-target strategies combining redox modulation with interventions that directly engage synaptic plasticity, neurotrophic signaling or critical developmental windows, to achieve functional behavioral outcomes in ASD.

## Supplementary Information

Below is the link to the electronic supplementary material.

Supplementary Material 1 (DOCX 159 KB)

## Data Availability

All data supporting the findings of this study are available within the paper and its Supplementary Information. This study does not include any gels or blots images.
