# Supplementary material for: PEGylated Liposomal Resveratrol Induces Region-Specific Redox Modulation Without Behavioral or Neurotrophin Recovery in a VPA Model of Autism
Source: J Mol Neurosci. 2026 Aug 1;76(3):121. doi: 10.1007/s12031-026-02574-1 (PMC13428714; doi:10.1007/s12031-026-02574-1)
Supplement: Supplementary file 1 — Supplementary Material 1 (DOCX 159 KB) [file 12031_2026_2574_MOESM1_ESM.docx]

**Hemocompatibility Test**

Hemolytic activity was assessed by incubating human erythrocytes with different liposomal formulations at four concentrations: liposomes (with or without the presence of PEG groups in the structure) at 6 mg/mL (100%), 3 mg/mL (50%), 1.5 mg/mL (25%), and 0.75 mg/mL (12.5%); and resveratrol at 1 mg/mL (100%), 0.5 mg/mL (50%), 0.25 mg/mL (25%), and 0.125 mg/mL (12.5%). Hemoglobin release was then quantified by spectrophotometry at 540 nm. Positive (distilled water) and negative (0.9% saline solution) controls confirmed methodological reliability, exhibiting complete hemolysis (100%) and minimal hemolysis (<1%), respectively, at all tested concentrations. It is important to note that the hemolysis threshold is 5%.

Formulations composed of PEG liposomes and PEG liposomes with RSV demonstrated good hemocompatibility, with hemolysis levels below 4% at the highest concentration and below 1% at lower concentrations (25% and 12.5%). The formulation containing PC combined with PEG liposomes showed significantly increased hemolysis in a dose-dependent manner. At 100% concentration, complete hemolysis was observed, which decreased to approximately 65% at 50%, 40% at 25%, and 10% at 12.5% (p < 0.05 compared to the other formulations).

The liposomal formulation containing PEG + PC + RSV significantly reduced hemolytic toxicity. Hemolysis in this group remained below 3% at all tested concentrations, like the values observed in the PEG liposome-only formulations (Supplementary Figure 1).

**
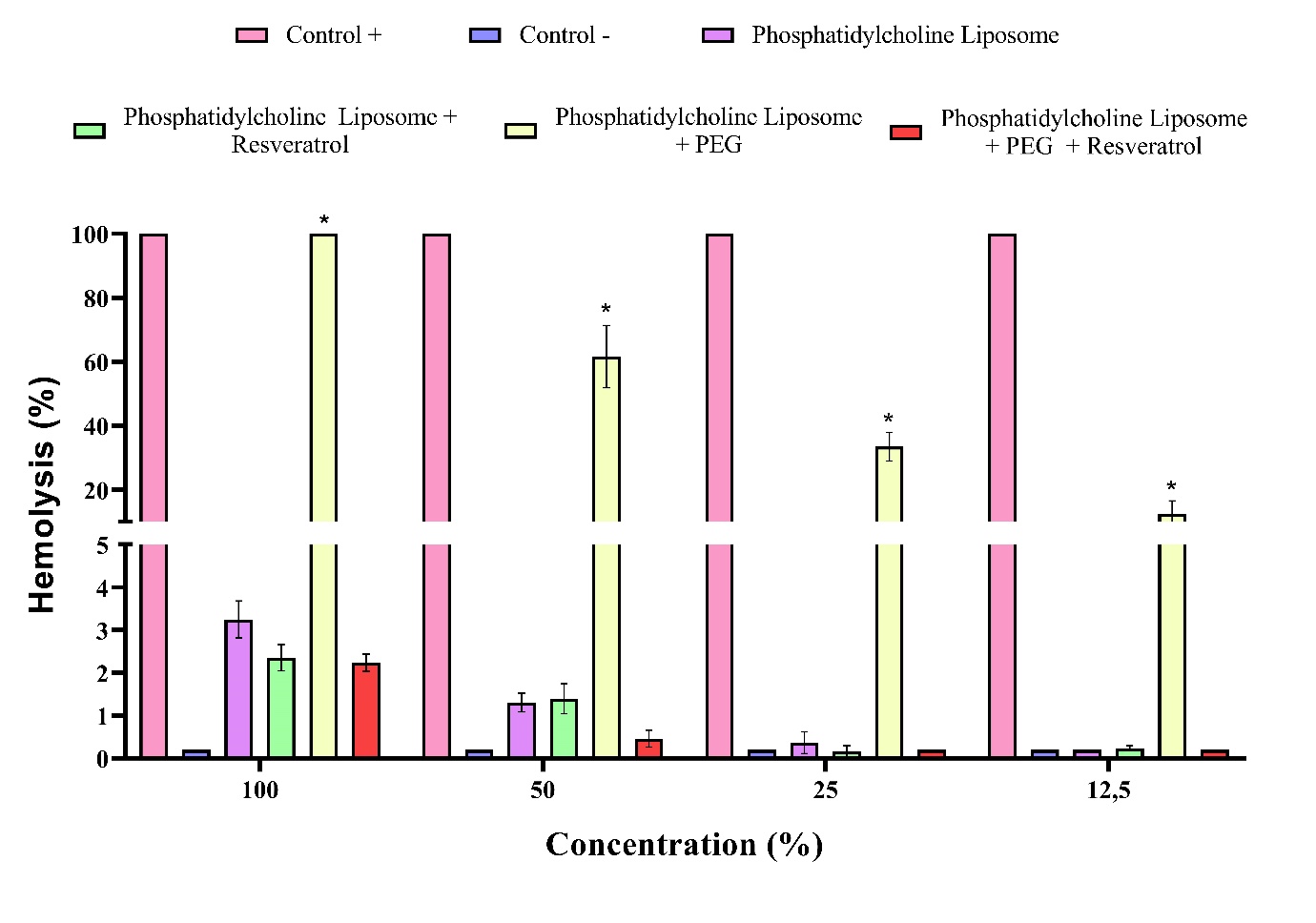
Supplementary Figure 1. Hemocompatibility test.** Evaluation of the hemolytic potential of liposomes at concentrations of 6 mg/mL (100%), 3 mg/mL (50%), 1.5 mg/mL (25%), and 0.75 mg/mL (12.5%), and resveratrol at concentrations of 1 mg/mL (100%), 0.5 mg/mL (50%), 0.25 mg/mL (25%), and 0.125 mg/mL (12.5%). Data are presented as mean ± SEM (n = 3). Statistically significant differences are indicated by * p < 0.001 compared to the negative control group (one-way ANOVA followed by Tukey’s post hoc test).
